# Two anchoring proteins control daughter apical complex assembly in *Toxoplasma gondii*

**DOI:** 10.64898/2026.02.13.705759

**Published:** 2026-02-14

**Authors:** Mélanie Burette, Marcin Pezinsky, Quynh Nguyen, Alae Benharoual, Sharon Patray, Marjorie Maynadier, Jason Delabre, Thomas Mouveaux, Arnault Graindorge, Laurence Berry, Jean-Francois Dubremetz, Valérie Rofidal, Yi-Wei Chang, Mathieu Gissot, Maryse Lebrun

**Affiliations:** 1 Laboratory of Pathogens and Host Immunity, LPHI, UMR 5294 CNRS, UA15 INSERM, Université de Montpellier, Montpellier 34095, France; 2 Univ. Lille, CNRS, Inserm, CHU Lille, Institut Pasteur de Lille, U1019-UMR 9017-CIIL-Center for Infection and Immunity of Lille, F-59000 Lille, France; 3 Department of Biochemistry and Biophysics, Perelman School of Medicine, University of Pennsylvania, Philadelphia 19104, USA; 4 Institute of Structural Biology, Perelman School of Medicine, University of Pennsylvania, Philadelphia 19104, USA; 5 IPSiM, Univ. Montpellier, CNRS, INRAE, Institut Agro, France

## Abstract

During *Toxoplasma gondii* division, the apical complex—comprising the conoid, apical polar ring (APR), and preconoidal rings—assembles with precise spatiotemporal coordination to form functional daughter buds. Despite their essential roles in invasion, motility, and division, the scaffolding proteins orchestrating this ordered assembly have remained largely unidentified. Here, we identify and characterize RCC1–2 and APR8 as essential factors directing distinct, sequential phases of daughter cell apical complex construction. Both proteins are recruited with precise spatial and temporal dynamics to the daughter buds, where they function as scaffolds rather than static structural components. APR8 transiently occupies the basal region of the APR specifically in early daughter cells. It is dispensable for conoid and PCR initiation, yet its loss causes APR collapse, abolishes SPMT anchoring, and eventually arrests conoid maturation. In contrast, RCC1–2 localizes beneath the APR basal layer and persists throughout daughter cell development, where it contributes to stabilizing the attachment of SPMTs to the APR. Notably, *in situ* cryo-electron tomography further reveals that the interspersed pillars bridging SPMTs ends to the APR fail to form properly in RCC1–2-depleted parasites. These findings map a hierarchical RCC1–2/APR8-dependent scaffolding process that advances our understanding of parasite replication.

## Introduction

*Toxoplasma gondii* is a protozoan parasite of significant clinical and veterinary importance. While most human carriers remain asymptomatic, the parasite can be a major threat to immunodeficient individuals. The pathogenesis of this unicellular organism primarily stems from the ability of its tachyzoite form to invade host cells, replicated intracellularly, and eventually egress, leading to cell death and tissue damage. This unicellular eukaryote exhibits a characteristic crescent shape with pronounced apico-basal polarity. Its morphology is maintained by an intricate cytoskeletal scaffold composed of the inner membrane complex (IMC), the underlying alveolin network, and 22 subpellicular microtubules (SPMTs)^1–3^. These microtubules radiate from a ring-shaped proteinaceous structure, called the apical polar ring (APR), and extend along roughly two-thirds of the parasite’s length^3,4^. The APR is further connected to a conical array of spiraling tubulin fibers, known as the conoid, which is capped by three preconoidal rings (PCRs)^5–8^. Within the conoid lie two short microtubules (intraconoidal microtubules, ICMTs) and two distinct classes of secretory organelles: the micronemes and rhoptries^1–3,8,9^. Collectively, the APR, PCRs, ICMTs, micronemes and rhoptries form the apical complex. The apical complex operates in tight coordination with the SPMTs and the inner membrane complex (IMC)—a set of double membrane sacs that lie beneath the parasite plasma membrane—to generate the mechanical force necessary for motility, host cell invasion, and egress^10–16^.

**Figure 1.**
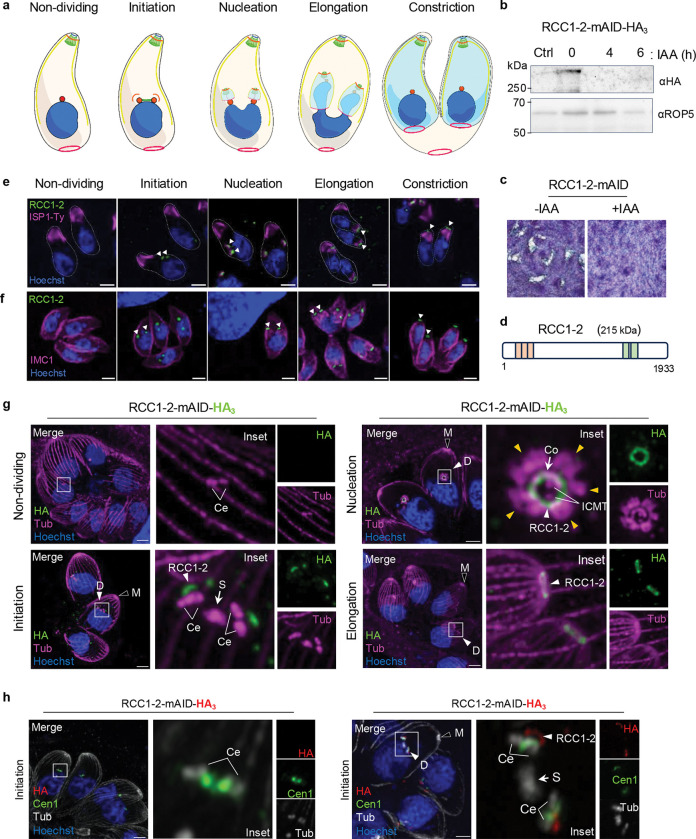
TgRCC1–2 is recruited at the apical end of daughter cells throughout endodyogeny. **a,** Schematic representation of *T. gondii* cell division by endodyogeny. The initiation step begins with centrosome duplication (red dot), followed by nucleation, during which DCS starts to assemble concurrently with the formation of nascent preconoidal ring (PCR; blue ring), conoid (green) and apical polar ring (APR; red ring). During the elongation step, the inner membrane complex (IMC, light blue) continues to elongate, and the nucleus (blue) adopts a characteristic U-shape prior to fission. In the final constriction step, the mother plasma membrane envelops the emerging daughter cells, while the residual maternal components are disassembled and degraded. **b,** Assessment of protein depletion in RCC1–2-mAID-HA_3_ cell lines by western blot following IAA treatment for 0, 4, or 6 hours. TgROP5 was used as loading control. **c,** Representative images of plaque assays for RCC1–2mAID-HA_3_ tagged line in the absence or presence of IAA. **d,** Schematic representation of the TgRCC1–2 protein, highlighting RCC1-repeats at the N-terminus (orange) and C-terminal coiled coil domains (green). **e,** Localization of RCC1–2 and ISP1 by confocal microscopy. Endogenously double tagged RCC1–2-mAID-HA_3_ and ISP1-Ty2 parasites were stained with anti-HA (green) and anti-Ty (magenta) antibodies to visualize RCC1–2 and the apical cap marker ISP1, respectively. DNA is labeled with Hoechst. White arrowheads point at RCC1–2 signal. Dotted outlines indicate individual parasites, with mothers shown in white and daughters in light blue. Shown are maximum-intensity projections of z-stack confocal images. Scale bars are 2μM. **f,** Confocal immunofluorescence images of RCC1–2-mAID-HA_3_ parasites, labeled with anti-HA (green) and anti-IMC1 (magenta) antibodies to visualize RCC1–2 and the IMC, respectively. DNA is labeled with Hoechst. White arrowheads point at RCC1–2 signal. Shown are maximum-intensity projections of z-stack confocal images. Scale bars are 2μM. **g**, U-ExM images of tachyzoites expressing RCC1–2 throughout endodyogeny. Parasites were stained with anti-HA (green) and anti-α/β tubulin (magenta) antibodies to label RCC1–2 and microtubules, respectively. Nuclear DNA was labeled with Hoechst (blue). Images represent maximum-intensity projections of z-stack confocal sections. Insets highlight daughter cell buds. Whitecontoured arrowheads indicate mother apical complex (M: mother). White arrowheads point developing daughter cells structures (D: daughter) (Ce: centrioles; S: spindle; ICMT: intraconoidal microtubules; Co: conoid). Scale bars are 5μM. Yellow arrowheads mark the five rafts of nascents SPMTs. **h,** U-ExM images of RCC1–2 in dividing parasites. Parasites were stained with anti-HA (red), anti-Centrin1 (green) and anti-α/β-tubulin (white) antibodies to label RCC1–2, centrioles and microtubules, respectively. Nuclear DNA was labeled with Hoechst (blue). Images represent maximum-intensity projections of z-stack confocal sections. Insets highlight centrosomes of mother cell. Black arrowheads indicate mother apical complex (M: mother). White-contoured arrowheads point developing daughter cells structures (D: daughter) (Ce: centrioles; S: spindle). Scale bars: 5μM.

The tachyzoites exhibit a simplified and rapid division mode called endodyogeny that produce two daughter cells within the mother cell (Fig. 1a). Tachyzoites exhibit a distinctive cell cycle featuring closed mitosis, consisting of three main phases—G1, S, and M—while the G2 phase is thought to be brief and to overlap with the S/M phase^17^. During the S and M phases, chromosome replication and organelle duplication occur in coordination with daughter cell development, culminating in cytokinesis that completes daughter cell formation. A single round of DNA replication and mitosis (nuclear cycle) is closely coordinated with the *de novo* formation of the daughter cell scaffold (DCS) in a process referred to as budding or budding cycle^3,18^. The bipartite nature of the centrosome enables the independent control of the nuclear (mitosis) and budding cycles^19^. It is only recently that the sequence of the DCS formation early events (budding initiation) have been discovered^18,20,21^. During endodyogeny, centrosome duplication marks the transition from G1 into the S phase and coincides with the beginning of the budding cycle^22,23^. Assembly of the DCS happens in close proximity to the duplicated centrioles, where both apical and basal poles of the daughter cells assemble concurrently^20,21,24^. A striated fiber assemblin (SFA) fiber links the nascent DCS to the centrosome^4,25^, coordinating spatial development of daughter cells. The earliest detectable event in DCS formation is the assembly of the APR which is oriented toward the centrioles and serves as an anchoring site for the cortical microtubules^20,21^. Soon after the nascent APR appears, both the forming conoid and the ICMTs assemble, accompanied by rafts of 5–6 SPMTs radiating from the APR^21^. Concurrently, the daughter IMC is assembled and elongates alongside the SPMT cytoskeleton^3^. As division progresses, cytoplasmic constriction culminates with the formation of two fully mature daughter cells, each containing a nucleus and organelles (Fig. 1a). In this process, the APR plays a pivotal role as the primary site of DCS formation. The APR has been proposed for many years as a critical anchoring site for the nucleation and organization of cortical microtubules. However, recent studies suggest that SPMT nucleation may instead originate at the centrioles where gamma tubulin is localized, with these microtubules subsequently translocated to the nascent APR^20,26^. In this context, the nascent APR is best viewed as a critical anchoring platform that stabilizes not only the SPMTs but also other apical structures, including the conoid, the ICMTs and IMC, thereby coordinating assembly at the apical pole^18,21^. The characterization of the structure and composition of the mature (mother) APR revealed three distinct concentric layers—upper, middle and bottom layers—each with a distinctive protein composition^7,16,27^. Whether these layers assemble in parallel or follow a defined temporal sequence during DCS formation remains unknown. Notably, despite this architectural complexity, none of the identified APR proteins have yet been found individually essential for DCS formation, indicating that key scaffolding components required to organize daughter bud remain to be discovered.

Here, we describe two critical scaffolding proteins, RCC1–2 and APR8, that govern distinct stages of DCS budding. APR8 is a bona fide component of the bottom APR layer. It is required for APR integrity and anchoring of SPMTs to APR, and is necessary to form the APR and conoid. RCC1–2 localizes just beneath the bottom layer of APR, defining likely a fourth layer of this complex structure, where it performs a more specific yet crucial scaffolding role by facilitating the proper anchoring and stabilization of SPMTs to the APR.

## RESULTS

### TgRCC1–2 is recruited at the apical end of daughter cells throughout endodyogeny

The Regulator of Chromosome Condensation 1 (RCC1) domain is a versatile module involved in the regulation of various cellular processes, including cell division^28^. In *Toxoplasma gondii* and other apicomplexans, RCC1-containing proteins remain largely uncharacterized, with the notable exceptions of Nd6^29^, MyoH^30^, MyoL^31^ and RCC1^32^, which have been implicated in rhoptry discharge, the PCR–conoid connection and motility, and nucleocytoplasmic transport and cell division, respectively. According to INTERPRO (IPR000408), 15 proteins are predicted to harbor an RCC1 domain in *Toxoplasma* (Supplementary Table 1). To investigate their potential roles, we tagged six of these proteins with a mini auxin-inducible degron (mAID) and a triple HA epitope, allowing conditional protein depletion upon addition of indole-3-acetic acid (IAA) and subcellular localization by immunofluorescence, respectively (Supplementary Fig. 1a–c). These candidates were selected based on their negative fitness scores^33^ and predominant expression during the S/M phase^34^, suggesting potential involvement in the formation of the apical complex and the regulation of its associated components.

We found two proteins, TGGT1_280770 (RCC1–2) and TGGT1_209130 (RCC1–6), present at the apical end of daughter cells, while the others were mostly diffuse in the cytoplasm of both non-dividing and dividing parasites (Supplementary Fig. 2a). Conditional downregulation of the proteins was achieved by addition of IAA to the culture medium. IFA and western blot analysis confirmed full depletion of proteins after 24 h of IAA treatment (Supplementary Fig. 2b, c). Only depletion of RCC1–2 affects the parasite lytic cycle as shown by complete absence of lysis plaques in monolayers of human foreskin fibroblasts (HFF) infected with RCC1–2-mAID strain after 7 days of auxin treatment (Fig. 1b, c; Supplementary Fig. 2c, d). RCC1–2 localization at the apical end of daughter cells and its requirement for sustaining the lytic cycle, strongly suggest that it might play an essential role during *Toxoplasma* division, prompting an in-depth characterization of the protein. RCC1–2 is a large protein of 215 kDa, containing three tandem RCC1 repeats in its N-terminal region and two coiled-coil domains in its C-terminal part (Fig. 1d; Supplementary Fig. 1a).

We first investigated the timing of RCC1–2 expression throughout the tachyzoite division process, using nuclear staining alongside two markers of IMC formation—ISP1 and IMC1—which track the formation of the apical cap and middle layer of daughter IMC, respectively. ISP1 is a well-established marker of the apical cap^35^—a distinctive, cone-shaped plate that forms the uppermost region of the IMC—which is physically connected to the APR. IMC1 staining was performed using specific anti-IMC1 antibodies, while for ISP1, the protein was C-terminally Ty-tagged in the RCC1–2-mAID-HA strain (Supplementary Fig. 3a, b) and detected using anti-Ty antibodies (Supplementary Fig. 3c). In non-dividing parasites, RCC1–2 signal is faint and appears highly diffuse in the cytoplasm (Fig. 1e). As parasites undergo division, two distinct puncta of RCC1–2 emerged in the cytoplasm of dividing cells in proximity to nucleus (Fig. 1e). This expression pattern was consistent with the S/M phase–specific expression profile of RCC1–2, according to ToxoDB data^36^. We found that RCC1–2 appears prior to the initiation of daughter IMC budding, as shown by the detection of the protein before the incorporation of ISP1 and IMC1 into nascent daughter cells (Fig. 1e, f). As the apical cap of the IMC begins to assemble, RCC1–2 localizes above the ISP1 signal (Fig. 1e) and remains confined at the apical pole of daughter cells until the complete daughter bud maturation; and eventually disappears from individualized newly daughter cells (Fig. 1e, f).

### RCC1–2 is recruited after centriole duplication but before SPMTs and conoid formation

We next performed ultrastructure expansion microscopy (U-ExM) to precisely determine the timing and subcellular localization of RCC1–2 in early developmental stages of DCS formation. To examine RCC1–2’s recruitment timing relative to key cytoskeletal structures, we stained α/β-tubulins which allows to visualize the mitotic spindle, the centrioles, the conoid and SPMTs. U-ExM analysis showed that RCC1–2 appears just after centriole duplication (Fig. 1g), a result confirmed by co-staining with centrin 1 marker (Fig. 1h). Initially, the SPMTs appear as discrete ‘rafts’—bundles composed of two to four individual SPMTs—positioned facing the centrioles during the early stage of DCS. RCC1–2 signal was systematically detected before the formation of microtubule rafts and conoid structure (Fig. 1g, initiation), suggesting its recruitment to the nascent DCS at a defined early stage of daughter cell biogenesis. At this early stage, RCC1–2 is detected as an arc near the centrioles—the opening of the arc facing the centriole region (Fig. 1g), reminiscent of the nascent APR^21^. Next, it adopted a ring-shaped structure, matching the size of the primordial conoid and is surrounded by the SPMTs raft (yellow arrowheads) (Fig. 1g, right panel). The transition from arc-shaped signal to a continuous ring coincides with the emergence of the conoid and the formation of the SPMT array. Notably, this ring-shaped structure consistently forms before the complete nucleation of all 22 cortical microtubules and is maintained until cytokinesis and individualization of daughter cells. RCC1–2 eventually disappears following cytokinesis. RCC1–2 is subsequently lost once cytokinesis is complete. We conclude that RCC1–2 is a daughter-specific protein recruited to the DCS at a very earliest stage of daughter bud initiation and until the end of the endodyogeny process.

### RCC1–2 localizes below the bottom layer APR

**Figure 2.**
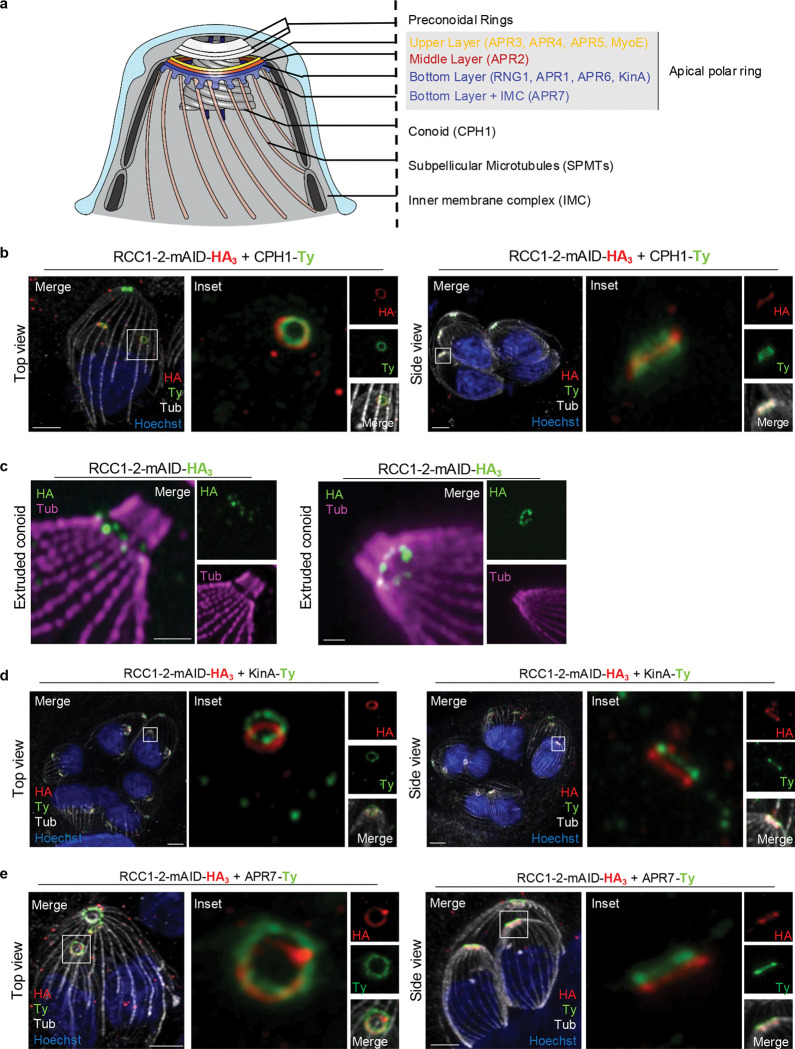
RCC1–2 localizes below the bottom layer APR. **a,** Schematic representation of *T. gondii* apical complex in retracted conoid state (adapted from Ren *et al.*^16^). **b,** U-ExM images of intracellular tachyzoites showing the localization of RCC1–2 (red), the Ty-tagged conoid protein, CPH1 (green) and α/β tubulin (white). Images represent maximum-intensity projections of z-stack confocal sections, with both top and side views are left and right panels, respectively. Insets highlight the apical complex of a single developing daughter cell. Scale bars are 5μM. **c,** U-ExM images of RCC1–2 localization and tubulin (magenta) in syringed-lysed tachyzoites with extruded conoid state. Images represent z-stack confocal sections. Scale bars are 1μM. RCC1–2 localized beneath the extruded conoid, extending into SPMTs interspaces. **d-e,** U-ExM images of intracellular tachyzoites showing the localization of RCC1–2, tubulin and Ty-tagged bottom layer APR proteins, KinA (**d**) and APR7 (**e**). Images represent maximum-intensity projections of z-stack confocal sections, with both top and side views shown (left and right panels, respectively). Insets highlight the apical complex of a single developing daughter cell. Scale bars are 5μM. RCC1–2 localized below the last known layer of the APR. b, d, e) DNA is labeled by Hoechst (blue).

To precisely localize RCC1–2 within the apical complex, we first endogenously C-terminally Ty-tagged the conoid protein hub 1 (CPH1)^11^ (Fig. 2a, Supplementary Fig. 3). During daughter bud, RCC1–2 localizes as a ring encircling the elongated conoid, approximately midway along its length (Fig. 2b, right panel). It should be noted that in intracellular parasites, the conoid is always retracted and the APR is not located at the conoid base, as observed when conoid is extruded in extracellular parasites. Given that RCC1–2 is exclusively expressed in daughter cells, it is challenging to precisely define the location of RCC1–2 when the conoid is extruded. To overcome this limitation, intracellular tachyzoites were extracted from infected host cells and subsequently treated with the calcium ionophore A23187 to induce conoid extrusion^37^. RCC1–2 signal was detected only in a few rare parasites. In these parasites, RCC1–2 localized beneath the extruded conoid (Fig. 2c), consistent with the localization pattern observed for APR proteins. Additionally, RCC1–2 appears closer to SPMTs than conoid base (Fig. 2c).

The APR itself is a fixed multilayered structure^7,8,16,27,38^, including protein rings such as apical polar ring APR3 (upper layer), APR2 (middle layer), APR1, Kinesin A (KinA) and APR7 (bottom layer), each localizing to specific subregions and conferring stability to the entire complex (Fig. 2a). APR7 extends beyond the APR staining reaching into the spaces between adjacent SPMTs, consistent with a localization at the interface between the APR and the minus ends of the SPMTs^16^. Because of the proximity of RCC1–2 with the SPMTs (Fig. 2c), we performed colocalization with the bottom layer proteins KinA and APR7 (description of endogenously C-terminally Ty-tagged lines in Supplementary Fig. 3). We first looked at early stage of division. As anticipated, the recruitment of RCC1–2 to the apical complex precedes that of KinA, which is known to be recruited following the formation of SPMT rafts^10^ (Supplementary Fig. 3d). The timing of APR7 recruitment to the DCS had not been investigated before. Here, we demonstrated that APR7 is recruited subsequent to RCC1–2, when the SPMT rafts are formed (Supplementary Fig. 3d). Remarkably, colocalization studies at later stages showed that RCC1–2 localizes beneath both KinA and APR7 (Fig. 2d, e), strongly supporting that RCC1–2 occupies a previously uncharacterized space below the bottom layer of APR defined by APR7.

### Conditional depletion of RCC1–2 causes structural defects of the pelicule

**Figure 3.**
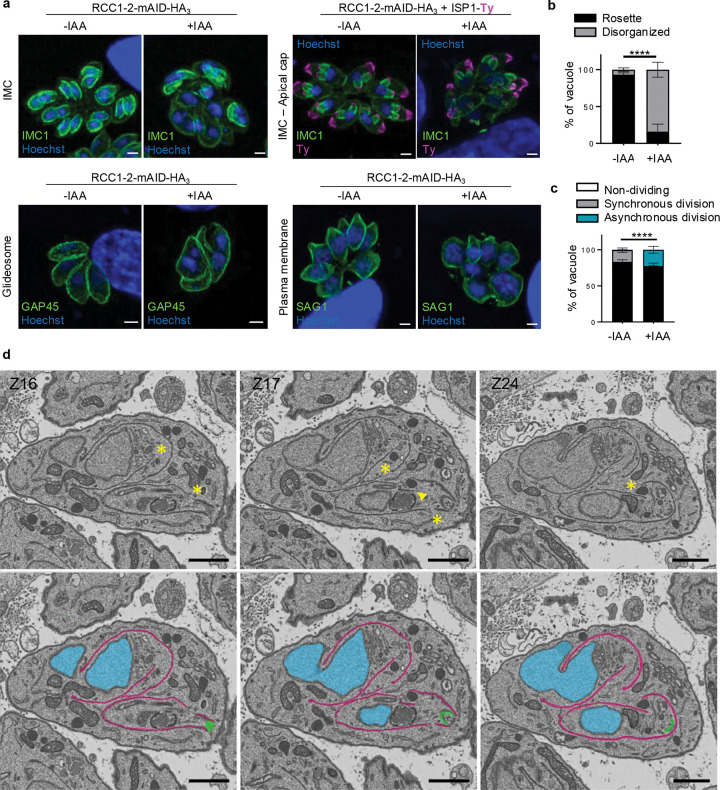
Loss of RCC1–2 causes structural defects of the pellicle. **a,** Confocal immunofluorescence images of RCC1–2-mAID-HA_3_ parasites costained with IMC1, GAP45 and SAG1 antibodies, or RCC1–2-mAID-HA_3_ parasites expressing ISP1-Ty2, left untreated or treated with IAA for 24h. DNA is labeled with Hoechst. Scale bars are 2μM. **b-c**, Quantification of phenotypical defects displayed in panel (a). **b,** Rosette and disorganized vacuoles were quantified from approximately 100 vacuoles and are presented as mean ± SD (n = 3 biological replicates). Statistical significance was determined by two-way ANOVA test. **c,** Non-dividing, synchronous and asynchronous dividing vacuoles were quantified from approximately 300 vacuoles and are presented as mean ± SD (n = 3 biological replicates). Statistical significance was determined by two-way ANOVA test. Vacuoles containing parasites at multiple division stages were scored as asynchronous. **d,** Selected electron micrographs from 70 nm serial sections of dividing RCC1–2-depleted tachyzoites. The section number is indicated at the top left corner. 1^st^ row: the IMC of the daughter cells showed multiple interruptions (yellow asterisks), partial overlapping of IMC sheets and isolated IMC fragments (yellow arrowhead). The second row shows the same image after segmentation of the cellular compartments. Daughter IMC (pink), nucleus (blue), conoid (green). Scale bars are 1 μm.

To elucidate the underlying cause of lethality in absence of RCC1–2, we first examined the morphology of RCC1–2-depleted parasites. We stained the parasites with markers of the pellicule and the glideosome: the IMC marker IMC1, the plasma membrane protein SAG1, and the glideosome component GAP45 (Fig. 3a). In RCC1–2-depleted parasites (24 hours IAA), all three markers displayed irregular and discontinuous staining patterns, in contrast to the uniform labelling observed in untreated parasites that maintain their characteristic morphology, indicating a strong impact on the organisation of the IMC and pellicule. In addition, multinucleated parasites were observed, indicating a failure in proper daughter cell formation. Notably, depletion of RCC1–2 disrupted the typical rosette organization of tachyzoites within parasitophorous vacuoles (Fig. 3a, b, Supplementary Movies 1 and 2). Labelling of the IMC with IMC1 and ISP1 further revealed that parasites were at different stages of budding (Fig. 3a), indicating loss of synchrony during endodyogeny. This asynchrony was consistently observed in nearly all vacuoles (Fig. 3c). Staining of the apical secretory organelles, marked by AMA1 for micronemes and ARO for rhoptries, showed that these organelles remained associated with the parasite apex without major differences with wild-type (Supplementary Fig. 4a, b).

To further investigate the consequences of TgRCC1–2 depletion, we analyzed the ultrastructure of RCC1–2-depleted tachyzoites using transmission electron microscopy (TEM). TEM of RCC1–2-mAID-HA parasites treated with IAA for 24h confirmed that the secretory organelles were correctly positionned to the apical end and that the conoid was readily identifiable in both the daughter and mother cells (Fig. 3d). The IMC of daughter cells appeared well positionned relative to the conoid however, on many occasions, it appeared interupted (Fig. 3d). Interruption of the IMC of the mother was also observed (Supplementary Fig. 4c). Together, these observations demonstrate that TgRCC1–2 is essential for proper pellicle organization and the coordinated progression of parasite division.

### RCC1–2 is required for proper anchoring of SPMTs to APR

**Figure 4.**
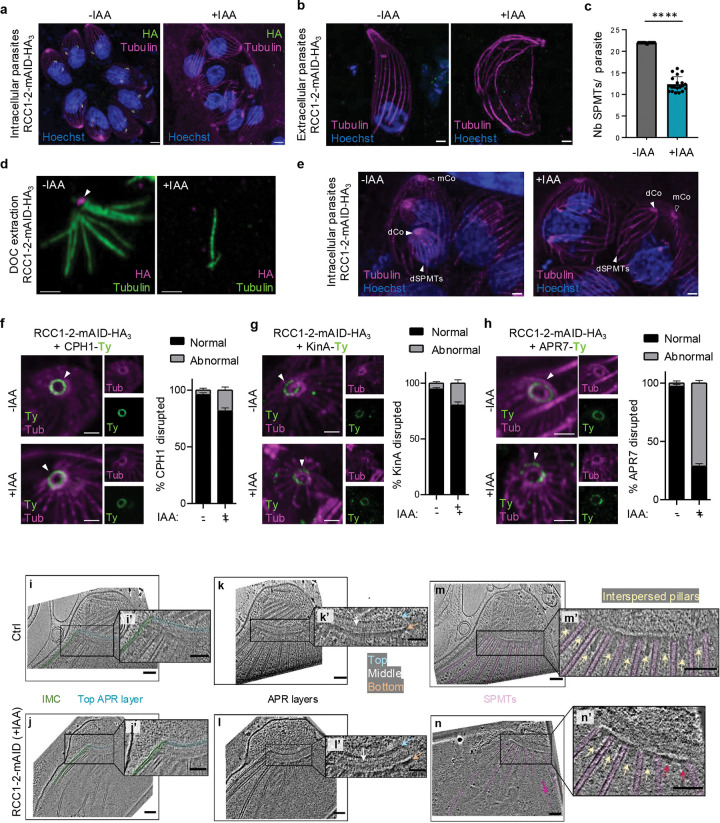
RCC1–2 is required for SPMTs anchoring to the APR. **a-c)** SPMTs organization in RCC1–2-depleted tachyzoites. U-ExM images of intracellular (a) or syringed-lysed tachyzoites (b) left untreated or treated with IAA for 24h. Tachyzoites were stained with anti-HA (green), anti-α/β tubulin antibodies (magenta) to label RCC1–2 and microtubules respectively. DNA is labeled with Hoechst (blue). Images represent maximum-intensity projections of z-stack confocal sections. Scale bars are 5μM. **c,** Quantification of SPMTs defects displayed in panel (**b**). SPMTs number was quantified from approximately 20 parasites and are presented as mean ± SD (n = 3 biological replicates). Statistical significance was determined by one-way ANOVA test. **d,** Images of freshly egressed parasites subjected to DOC extraction. Parasites were labeled with an anti-HA (magenta) and anti-acetylated tubulin (green) antibodies to label RCC1–2 and microtubules, respectively. Scale bars are 2μM. **e,** U-ExM images of dividing tachyzoites, either left untreated (left panel) or IAA-treated (right panel) for 24h. Tachyzoites were stained with anti-α/β tubulin antibodies (magenta) to label microtubules and Hoechst (blue) for DNA. Images represent maximum-intensity projections of z-stack confocal sections. White-contoured arrowheads indicate mother conoid (mCo). White arrowheads point daughter cells structures (dCo: daughter conoid; dSPMTs: daughter subpellicular microtubules). Scale bars are 2μM. **f-h,** Representative images of apical markers in daughter cells by U-ExM. U-ExM images of RCC1–2-mAID-HA_3_ + CPH1-Ty2 **(f)**, + KinA-Ty2 **(g)** and + APR7-Ty2 **(h)** parasites, either left untreated or treated with IAA for 24h. Intracellular tachyzoites were stained with anti-Ty (green) and anti-α/β tubulin (magenta) antibodies to label Ty-tagged proteins and microtubules, respectively. Images represent maximum-intensity projections of z-stack confocal sections. White arrows indicate apical complex of daughter cells. Disruption of the targeted marker was quantified from approximately 60 parasites and are presented as mean ± SD (n = 3 biological replicates). Statistical significance was determined by two-way ANOVA test. Scale bars are 1μM. **i-n,** Cryo-ET tomograms of control and RCC1–2-depleted parasites. (**i-j**) Tomogram slices of WT (i) and RCC1–2-depleted parasites (j) both show the inner membrane complex (IMC, green) anchored to the top layer of the apical polar ring (APR, blue), as detailed in the enlarged insets (i’, j’). (**k-l**) Tomogram slices of the apical end show that the top (blue arrow), middle (white arrow), and bottom (orange arrow) layers of the APR exhibit band-like appearance in both WT (k, k’) and RCC1–2-depleted parasites (l, l’). (**m-n**) Tomogram slices showing SPMT attachment sites on APR in WT (m, m’) and RCC1–2-depleted (n, n’) parasites. SPMTs (magenta); SPMT detached from the APR (magenta arrow); interspersed pillars densities franked by attached SPMTs (yellow arrows); interspersed pillars densities next to the detached SPMTs from the APR (red arrows). Scale bars: 100 nm

Considering that the organization of the IMC relies on the underlying SPMTs and that RCC1–2 is present at the bottom part of the APR, where SPMTs are anchored, we tested whether the global SPMT architecture was also perturbed upon RCC1–2 depletion. Intracellular RCC1–2-mAID-HA parasites treated for 24 h with IAA were processed by U-ExM and SPMTs were stained with anti-α/β-tubulin antibodies. In RCC1–2-depleted parasites, SPMTs were still formed, showing that RCC1–2 does not play a role in tubulin nucleation; however, the symmetry and organization of SPMTs in mother cells was disrupted (Fig. 4a). Parasites lacked the full complement of 22 SPMTs (Fig. 4b; Supplementary Fig. 5a, Supplementary Movies 3 and 4). Three-dimensional reconstruction of extracellular parasites and quantification of SPMTs showed an average of 12 per parasite in absence of RCC1–2 (Fig. 4b, c; Supplementary Fig. 5a), suggesting improper anchoring of SPMTs to the APR. To support this hypothesis, we performed sodium deoxycholate (DOC) extraction on freshly egressed parasites, followed by immunofluorescence to visualize the microtubules. In this experiment, both plasma membrane and IMC membranes are removed by detergent extraction, while the IMC network and the underlying cortical microtubules remain intact and anchored to the conoid via the APR. In absence of IAA, RCC1–2 remained associated with the SPMTs in DOC-extracted parasites (Fig. 4d), showing that RCC1–2 is associated with cytoskeleton structure. In contrast, we found a complete disassembly of the cytoskeleton in RCC1–2-depleted parasites, with only individual microtubules visible by IFA (Fig. 4d), indicating that RCC1–2 plays a crucial role in proper anchoring SPMTs to apical complex. We next examined the impact of RCC1–2 depletion on the SPMTs of developing daughter cells. In the absence of RCC1–2, the shape of the SPMTs was noticeably altered in daughter buds (Fig. 4e, Supplementary Movies 5 and 6). MORN1, which localizes to the basal end of daughter cells^39^, is well positionned in the mutant (Supplementary Fig. 6a, Supplementary Movies 5 and 6), but its ring structure was often irregular or appeared as discontinuous segments (Supplementary Fig. 6b). TEM analysis showed that the basal complex retained its typical architecture, defined by an electron-dense ring at the basal end of forming daughters (Supplementary Fig. 6c), supporting that the mishaped basal complex ring structure upon RCC1–2 depletion likely results from disrupted structural support provided by the IMC and SPMTs.

Together, these observations indicate that RCC1–2 is crucial for maintaining the proper architecture of SPMTs in developing daughter cells. Because RCC1–2 is present only in daughter cells and absent from mature parasites, these findings support its role as a scaffolding protein that recruits factors required to preserve SPMTs anchoring to the APR in mature parasites. As expected, the associated morphological defects markedly impaired parasite gliding motility (Supplementary Fig. 5c) and host cell invasion (Supplementary Fig. 5d). Nevertheless, although RCC1–2-depleted parasites were able to lyse the parasitophorous vacuole (Supplementary Fig. 5e), their severe motility defect prevented them from spreading to adjacent host cells, as shown by the egress assays (Supplementary Fig. 5f).

We next examined the impact of RCC1–2 depletion on the conoid and APR architectures. U-ExM and IFA analyses were performed on RCC1–2-mAID-HA_3_ parasites expressing Ty-tagged CPH1, KinA, or APR7, in absence or presence of IAA for 24 hours. In RCC1–2-depleted parasites, the conoid marker CPH1 and the APR markers KinA and APR7 consistently remained localized apically in daughter cells (Fig. 4f–h). The conoid ring structures remained largely intact following treatment, as visualized by fluorescence microscopy (Fig. 4e) and confirmed by TEM (Fig. 3d). KinA staining also appeared preserved overall (Fig. 4g). However, although APR7 was still detected in daughter cells following 24 hours of IAA treatment, its ring structure appeared interrupted in approximately 71% of vacuoles (Fig. 4h), a phenotype similarly observed in mother cells (Supplementary Fig. 5b). Both KinA and APR7 localize to the bottom layer of the APR, yet APR7 extends vertically beyond this layer, likely reaching the interspace between the SPMTs^16^. The severe disruption of the APR7 ring in the absence of RCC1–2, combined with the positioning of RCC1–2 immediately beneath APR7 and the pronouced defect in SPMTs anchoring to the apical complex, supports a role for RCC1–2 in organizing the linkage between SPMTs and the APR rather than structuring the APR itself.

To investigate the ultrastructural basis of these defects, RCC1–2 depleted parasites were subjected to cryo-electron tomography (cryo-ET). Following RCC1–2 depletion, the IMC-APR attachment remained morphologically indistinguishable from wild-type (Fig. 4i, i’, j, j’). We also observed focal breakage and loss of structural continuity along the IMC in parasites with intact PM (Supplementary Fig. 4d). Tomogram slices of the apical end showed an overall preserved APR in the mutant: the top, middle and lower layers of the APR retained their band-like appearance in both wild-type (Fig. 4k, k’) and RCC1–2-depleted parasites (Fig. 4l, l’). Remarkably, while wild-type parasites showed organized anchoring of the SPMTs to the APR (Fig. 4m, m’), depletion of RCC1–2 resulted in dissociation of some SPMTs from the APR (Fig. 4n, n’), corroborating the results observed by U-ExM. Interestingly, the basal projections sandwiched between adjacent SPMTs and connected to the bottom APR layer (named amorphous APR-associated density [AAD]^7,27^ or interspersed pillars^40^) seemingly exhibit variable disruptions (Fig. 4n’, yellow arrows), with the ones next to detached SPMTs showing the most reduced densities (Fig. 4n’, red arrows). Measurements of interspersed pillars were obtained from two wild-type and two RCC1–2-depleted parasites. The mean of interspersed pillars length in wild-type parasites was 70 nm (N=15), whereas RCC1–2-depleted parasites exhibited a reduced mean length of 51 nm (N=16). At sites of microtubule disruption, the average of interspersed pillars length on either side of the broken microtubules was further shortened to approximately 30 nm (N=4). This basal projections, attached to one side of the end of the SPMTs ^27^ are proposed to function as a linker for the SPMTs, APR and IMC. Together with the abnormal shape of APR7 ring and improper SPMTs anchoring, these results support a role of RCC1–2 in shaping the connection between SPMTs and APR.

### RCC1–2 proximity labeling reveals new daughter-specific proteins

**Figure 5.**
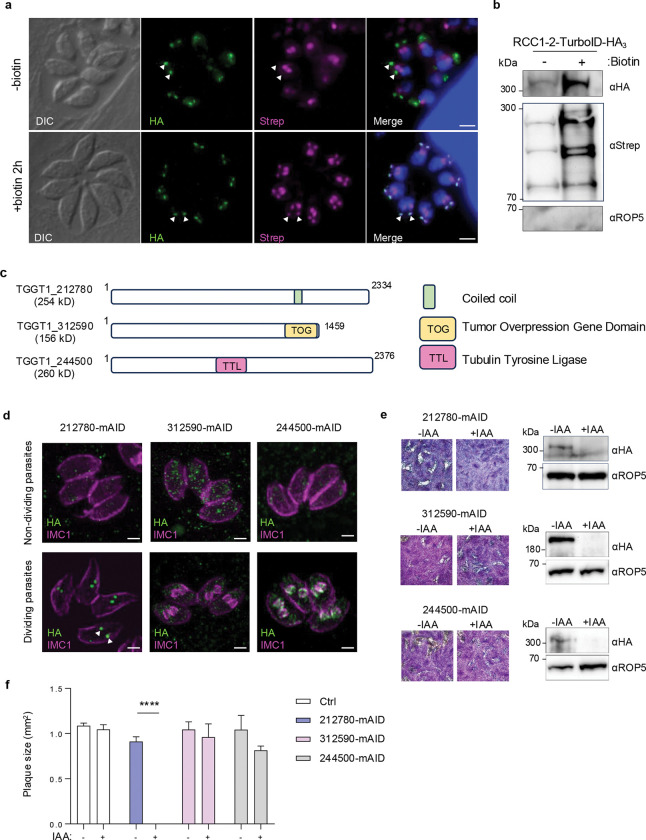
Proximity labelling of RCC1–2 identifies novel daughter-specific proteins. **a,** Confocal immunofluorescence images of RCC1–2-HA3-TurboID parasites after 0 or 2 hours of biotin treatment. White arrowheads point at RCC1–2 signal. Parasites were labeled with anti-HA (green) and streptavidin-based fluorophore (magenta) to label RCC1–2 and biotinylated sites, respectively. Scale bars are 2μM. **b,** Western blot analysis of immunoprecipitated proteins with anti-streptavidin beads from RCC1–2-HA_3_-TurboID parasites untreated or treated with biotin for 2 hours. Samples were immunobloted with anti-HA (upper panel), anti-streptavidin-HRP (middle panel). TgROP5 was used as IP negative control (lower panel). **c,** Schematic representation of the size of the three RCC1–2 proximity candidates and their INTERPRO-annotated functional domains^51^. Coiled-coil domain (green). TOG: Tumor Overexpression Gene,. TTL: Tubulin Tyrosine Ligase domain. **d,** Confocal immunofluorescence images of the three candidate proteins fused with a mAID-HA_3_ cassette (-mAID for short) in non-dividing (upper panel) and dividing parasites (lower panel). Parasites were stained with anti-HA (green) and anti-IMC1 (magenta) antibodies to visualize HA-tagged proteins and the IMC, respectively. White arrowheads point at apical signal. Scale bars are 2μM. **e-f,** Assessment of fitness and protein depletion of the three retained candidates. Immunoblot analysis (**e**, right panel) using an anti-HA antibody was performed on lysates from intracellular parasites (mAID-HA tagged lines) that were either untreated or treated with IAA for 24h. TgROP5 was used as loading control. Images of plaque assay (**e**, left panel) and quantification of plaques areas (**f**) for control (Tir1) and mAID-HA_3_ tagged lines in the absence or presence of IAA. Values are reported as mean ± SD (n = 3 biological replicates, each with three technical replicates). Statistical significance was determined by unpaired two-tailed Student’s t-test. RCC1-proximity candidates were rapidly degraded upon addition of IAA and depletion resulted in no visible plaque formation after 7 days only for TGGT1_212780.

RCC1–2 is associated with cytoskeletal structures and largely insoluble (Fig. 4d, Supplementary Fig. 7c), which prevents the identification of potential direct partners by immunopurification. Instead, to discover new proteins that, like RCC1–2, function at early stages of the budding process, we employed the TurboID biotin ligase proximity labeling approach^41^. A triple HA-tagged TurboID biotin ligase construct was fused to the endogenous C-terminal locus of RCC1–2 (Supplementary Fig. 7a, b). To first assess whether RCC1–2-HA3-TurboID is enzymatically active, intracellular parasites were incubated with or without biotin for 2 hours and IFAs were performed using fluorophore-conjugated streptavidin to label biotinylated proteins and anti-HA antibodies to localize RCC1–2. In the absence of biotin, streptavidin labelling was restricted to the apicoplast, which contains endogenous biotin (Fig. 5a). Upon biotin supplementation, however, an additional streptavidin signal colocalized with RCC1–2-HA3-TurboID at the DCS (Fig. 5a), confirming the enzymatic activity of RCC1–2-HA3-TurboID. These results provided the basis for subsequent immunopurification and identification by mass spectrometry of proteins located in close proximity to RCC1–2. Intracellular parasites were treated with a DOC-based cytoskeletal lysis buffer and biotinylated proteins were captured by immunoprecipitation using streptavidin-coated magnetic beads followed by mass spectrometry analysis. Western blot analysis showed a marked increase in biotinylated proteins in presence of biotin (Fig. 5b, Supplementary Fig. 7c). A total of 460 proteins were identified with a marked enrichment of proteins associated with the apical, and basal complexes (Supplementary Table 2). The identified apical complex proteins include components of the APR such as APR1, RNG2 and KinA^10,42^. Most of known basal complex proteins were enriched (BCC0, BCC2, BCC3, BCC4, BCC5, BCC6, BCC7, BCC10, BCC11, FIKK, MyoJ^24,43–45^). Among enriched proteins are also α and β tubulins, many proteins of the γ-tubulin complex (GCP2, GCP3, GCP4 and GCP5)^20,26,36^, but also many apical cap proteins (AC2, AC7, AC8, AC10, AC12^12,36,46,47^) and some IMC proteins such as IMC29^48^, IMC30^48^, IMC33^47^ and IMC43^49^ that are recruited at the early bud during endodyogeny. This comprehensive proteomic profiling aligned well with the early location of RCC1–2 to DCS and with previous studies showing coordinated development of the IMC, SPMTs, apical and basal complexes during daughter cell assembly in *Toxoplasma*.

To select the most relevant RCC1–2-proximal candidates associated with DCS for characterization, we applied selection criteria based incorporating their enrichment in the TurboID assay, low fitness-conferring scores indicating essentiality^33^ and cyclical expression profiles peaking in the S/M phase of the cell cycle^34^. Furthermore, to specifically seek for proteins transiently expressed at the daughter bud, we prioritized candidates with uncharacterized localization patterns in the hyperLOPIT dataset^50^. Transiently expressed proteins during endodyogeny are indeed usually not found in the spacial proteomic analysis, as exemplified for RCC1–2. This combined approach enabled us to refine the list to proteins most likely involved in early DCS assembly and function. Among the 11 uncharacterized proteins thus filtered (Supplementary Table 3), we independently characterized two in a separate study—TGGT1_293170 and TGGT1_255700—and showed that both are associated with a fiber structure that colocalises with SFA (unpublished, manuscript in preparation) and play a role in daughter cell formation, thereby validating our approach. Bioinformatics analysis using INTERPRO^51^ and HHPRED^52^ detected a tumor overexpression gene (TOG) domain for TGGT1_312590 and a Tubulin Tyrosine ligase (TTL) domain for TGGT1_244500, which is predicted to be a tubulin polyglutamylase (Fig 5c). Notably, both TOG and TTL domains exhibit functional links to tubulin by interacting directly with tubulin and playing critical roles in regulating microtubule dynamics^53,54^. Interestingly, like RCC1–2, many of these proteins contain a coiled-coil segment (Supplementary Table 3).

We next investigated the localization and function of TGGT1_212780, TGGT1_312590 (TOG-containing protein) and TGGT1_244500 (Tubulin Tyrosin ligase) by endogenously tagged them with the mini auxin-induced degron (mAID) and a triple HA tag (Supplementary Fig. 7d). IFA revealed that TGGT1_312590 exhibited faint diffuse cytoplasmic distribution in both non-dividing and dividing parasites, while the expression of the other two proteins was particularly enriched to dividing parasites (Fig. 5d). The Tubulin Tyrosin ligase TGGT1_244500 was recruited to the daughter IMC (Fig. 5d). TGGT1_212780 displayed a localization pattern characterized by two puncta in parasites initiating division, similar to RCC1–2 and few APR proteins (Fig. 5d). Next, we assessed the ability of the mutants to complete the lytic cycle upon depletion of the proteins by adding IAA in the culture media. Depletion of the proteins was efficient for all transgenic lines, as shown by western blot (Fig. 5e) and IFA (Supplementary Fig. 7e) analysis after 24 h of auxin treatment. No significant reduction in plaque area was observed for the Tubulin Tyrosin ligase and TGGT1_312590 mutants (Fig. 5e, f) in monolayers of HFF cells, after 7 days of auxin treatment. In contrast TGGT1_212780-mAID failed to form lysis plaques (Fig. 5e, f). Based on its localization and essential role, we thus further characterized TGGT1_212780.

### TGGT1_212780 associated with the bottom layer of APR, and named APR8

**Figure 6.**
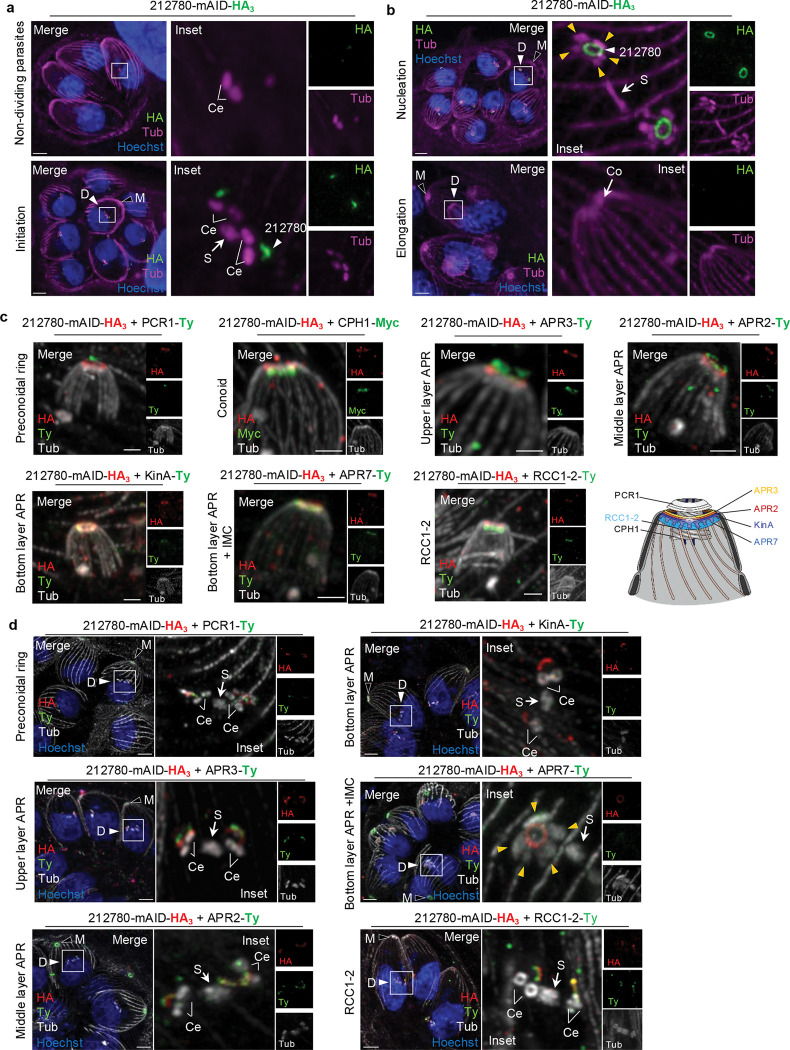
TGGT1_212780 is one of the earliest proteins expressed at the bottom layer APR during endodyogeny. **a,b** U-ExM images of dividing tachyzoites expressing TGGT1_212780-mAID-HA_3_ during endodyogeny. Parasites were stained with anti-HA (green) and anti-α/β tubulin (magenta) antibodies to label TGGT1_212780 and microtubules, respectively. Nuclear DNA was labeled with Hoechst (blue). Images represent maximum-intensity projections of z-stack confocal sections. Insets highlight developing daughter cells. White-contoured arrowheads indicate mother apical complex (M: mother). White arrowheads point developing daughter cells structures (D: daughter) (Ce: centrioles; S: spindle; Co: conoid). Yellow arrowheads mark the five rafts of nascents SPMTs. Scale bars are 5μM. **c,** U-ExM images of TGGT1_212780-mAID-HA_3_ in combination with multiple Ty/Myc-tagged apical complex proteins, shown as side view of a single daughter cell. Intracellular tachyzoites were stained with anti-HA (red), anti-Ty or anti-Myc (green) and anti-α/β tubulin (white) antibodies to label TGGT1_212780, apical markers and microtubules, respectively. Images represent maximum-intensity projections of z-stack confocal sections. Scale bars are 1μM. Bottom right: schematic showing apical complex markers in daughter cell: the PCR marker PCR1, the conoid protein CPH1 and the multilayered APR (from top to bottom: APR3, APR2, KinA, APR7 and RCC1–2). TGGT1_212780 is a daughter APR protein associated with the bottom layer of the APR. **d,** U-ExM images showing TGGT1_212780-mAID-HA_3_ in combination with multiple Ty-tagged apical complex proteins at early stage of endodyogeny. Intracellular tachyzoites were stained with anti-HA (red), anti-Ty or anti-Myc (green) and anti-α/β tubulin (white) antibodies to label TGGT1_212780, apical markers and microtubules, respectively. Nuclear DNA was labeled with Hoechst (blue). Images represent maximum-intensity projections of z-stack confocal sections. Insets highlight developing daughter cells. Black arrows indicate mother apical complex (M: mother). White-contoured arrowheass point developing daughter cells structures (D: daughter) (Ce: centrioles; S: spindle; Co: conoid). Yellow arrowheads mark the five rafts of nascents SPMTs. Scale bars are 5μM.

TGGT1_212780 can be detected as soon as parasites initiate division, presenting as two distinct puncta near the nucleus prior to the formation of cytoskeleton structures (Fig. 6a, Supplementary Fig. 8a), subsequently localizing to the apical end of DCS (Fig. 6b). Notably, TGGT1_212780 signal disappears very early, as the daughter IMC begins to elongate (Fig. 6b; Supplementary Fig. 8a), appearing to be much more transiently present in daughter cells compared to RCC1–2, which remained until cytokinesis (Fig. 1e, 1f). As observed for RCC1–2, TGGT1_212780 initially localizes as an arc positioned above the newly duplicated centrosomes (Fig. 6a), and then transitions into a complete ring structure at the apical pole of daughter cells (Fig 6b).

In order to precisely determine the localization of TGGT1_212780 within the apical complex, we co-labeled TGGT1_212780 with: i) preconoidal marker (PCR1)^14^, ii) conoid marker CPH1^11^, iii) APR markers (upper layer APR3; middle layer APR2; bottom layer KinA and APR7)^16^, iv) striated fiber SFA2^25^, and v) the newly described RCC1–2 protein. All the proteins were endogenously tagged with either a Ty or a Myc tag (Supplementary Fig. 8b, c). Examining both side-view (Fig. 6c) and top-view (Supplementary Fig. 8d) images of the colocalization of TGGT1_212780 with CPH1 and PCR1 showed that TGGT1_212780 localized beneath the PCRs but above the conoid, consistent with an APR localization. Colocalization analysis with the distinct APR layers revealed that TGGT1_212780 localized at a lower level than APR3 and APR2, while showing complete colocalization with KinA and partial overlap with APR7 at the bottom layer (Fig. 6c, Supplementary Fig. 8e). TGGT1_212780 was clearly localized above RCC1–2 (Fig. 6c, Supplementary Fig. 8e) and encircled the SFA2-labeled striated fiber, which connect the apical complex to the centrosome in daughter cells (Supplementary Fig. 8f). Based on this specific localization pattern, we concluded that TGGT1_212780 is a daughter APR protein associated with the bottom layer of the APR. According to the current annotation of APR proteins, TGGT1_212780 was hereafter designed APR8.

### Layered organization of the APR is stablished early during DCS

The multilayered organization of the APR has been characterized in mature parasites, but the timing of its assembly during DCS has not yet been investigated. Our colocalization and U-ExM analyses focusing on daughter cells provided the opportunity to address this question. Colocalization revealed that APR8 is incorporated earlier than KinA and APR7, while its characteristic arc-shaped signal appears concomitantly with APR2 (middle APR layer), APR3 (upper APR layer), and the PCR marker PCR1 (Fig. 6d). At early stage of endodyogeny, the signals for APR2, APR3, and PCR1 were already clearly resolved as distinct structures from APR8. Moreover, APR8 appears positioned below both APR2 (middle layer) and APR3 (upper layer), consistent with its localization in the bottom layer of the APR and supporting the idea that the three layers are established very early during DCS, before the emergence of cytoskeletal structures. In addition, APR8 and RCC1–2 were detected at the same time in the DCS, with the APR8 arc positioned above the RCC1–2 signal (Fig. 6d). Together, these observations indicate that PCR and APR assemblies are established concomitantly and that their respective layers are already segregated at the onset of bud initiation, with APR8 representing an early marker of the basal APR layer.

### APR8 acts as a scaffold for APR organization and subsequent proper apical complex development

**Figure 7.**
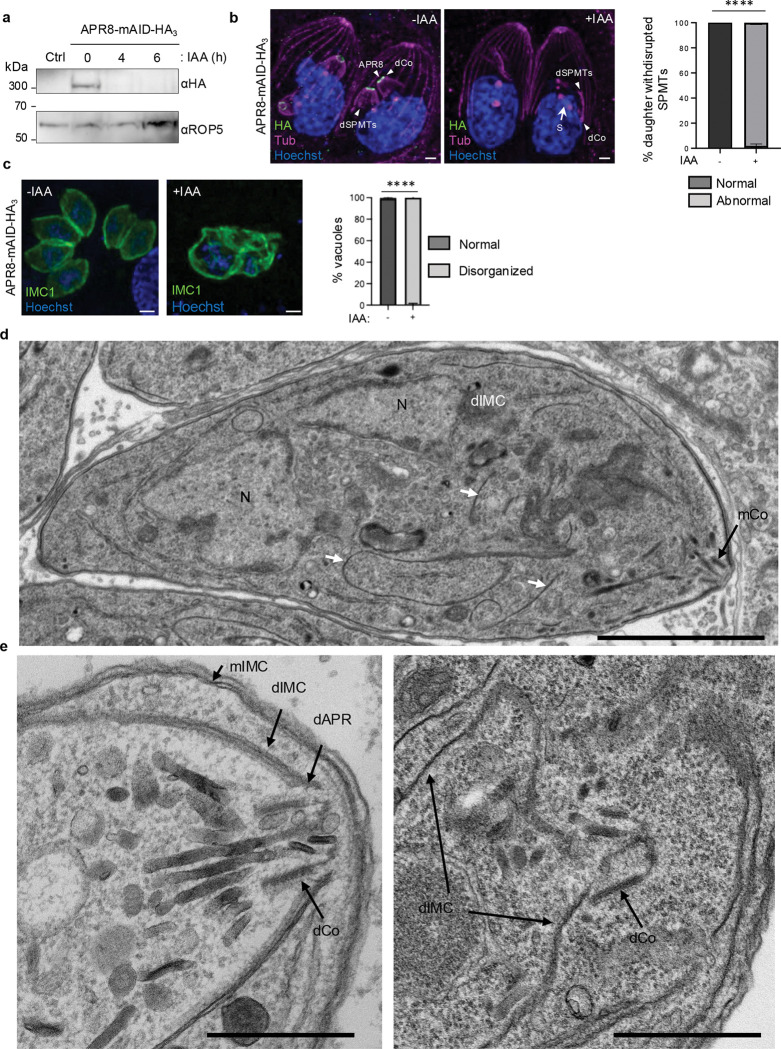
APR8 depletion prevents correct assembly of IMC and SPTMs. **A,** Western blot showing protein depletion in APR8-mAID-HA_3_ parasites following IAA treatment for 0, 4, or 6 hours. TgROP5 was used as loading control. APR82 was already undetectable within 4 h of IAA. **b-c,** SPMTs organization in APR8-depleted tachyzoites. U-ExM images of dividing tachyzoites, either left untreated (left panel) or IAA-treated (right panel) for 6h. Tachyzoites were stained with anti HA (green), anti-α/β tubulin antibodies (magenta) to label APR8 and microtubules, respectively. Nuclear DNA was labeled with Hoechst (blue). Images represent maximum-intensity projections of z-stack confocal sections. White-contoured arrowheads indicate mother conoid (mCo). White arrowheads point daughter cells structures (dCo: daughter conoid; dSPMTs: daughter subpellicular microtubules; S: spindle). Scale bars are 2μM. **c,** Quantification of SPMTs defects in daughter cells displayed in panel (**b**). SPMTs disruption was quantified from approximately 80 daughter cells and are presented as mean ± SD (n = 3 biological replicates). Statistical significance was determined by two-way ANOVA test. **c,** Confocal immunofluorescence images of the IMC in APR8-mAID-HA_3_ parasites, either left untreated or treated with IAA for 24h. Intracellular tachyzoites were stained with anti-IMC1 (green) and Hoechst (blue) to label the IMC and nuclear DNA, respectively. Scale bars are 2μM. Disruption of the IMC was quantified from approximately 100 vacuoles and are presented as mean ± SD (n = 3 biological replicates). Statistical significance was determined by two-way ANOVA test. **d-e,** Representative transmission electron microscopy (TEM) images of APR8-depleted tachyzoites. Panel **d** shows the whole mother cell upon endodyogeny while panel **E** focuses on daughter cells. White arrows highlight discontinuous flotting IMC of daughter cells (dIMC: daughter IMC; mIMC: mother daughter cell; N: nucleus). Scale bars: 2um (d), 0,5um (e).

To dissect the role of APR8 in early steps of the DCS formation, parasites were allowed to replicate for 24 hours and either left untreated or treated with IAA for 6 hours which represent the time needed to complete their cell cycle. Complete depletion of APR8 was observed after 4 hours of treatment (Fig. 7a). After 6 hours of incubation with IAA, only the SPMTs of the daughter cells are affected; 98% of daughter cells showing a severely disorganized cytoskeleton by U-ExM (Fig. 7b). At 24h, nearly all vacuoles exhibiting a distorted IMC architecture adopting a swollen shape (Fig. 7c). TEM of intracellular APR8-mAID-HA parasites incubated for 6 hours with IAA confirmed the important perturbation of the daughter cell IMC, that appears as discontinuous structure flotting in the cytoplasm of the mother cell (Fig. 7d, e). These finding showed that assembly of both the daughter IMC and SPMTs are not properly performed in absence of APR8.

**Figure 8.**
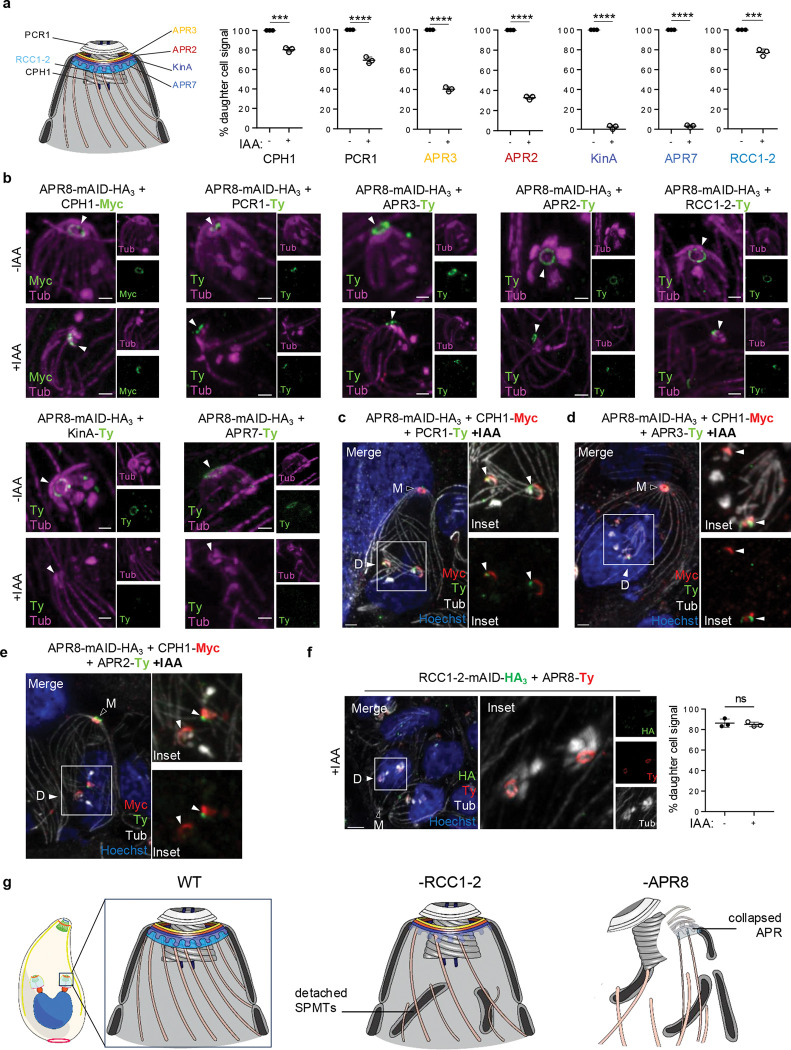
APR8 acts as a scaffold for APR organization and subsequent proper apical complex development. **a-b,** Apical markers expression in APR8-mAID-HA_3_ strain. **a,** Left: Schematic showing apical complex markers quantified in daughter cell: the PCR marker PCR1, the conoid protein CPH1 and the multilayered APR (from top to bottom: APR3, APR2, KinA, APR7 and RCC1–2). Right: Quantification of apical markers in APR8-mAID-HA_3_ in combination with Ty-/Myc-tagged apical markers, left untreated or IAA-treated for 6h. Presence of apical markers was quantified from approximately 50 daughter cells exhibiting elongated SPMTs and are presented as mean ± SD (n = 3 biological replicates). Statistical significance was determined by unpaired two-tailed Student’s t-test. **b,** Representative U-ExM images of daughter apical complex in APR8-mAID-HA_3_ strain with Ty-/Myc-tagged apical markers, either left untreated or IAA-treated for 6h Intracellular tachyzoites were stained with anti-Ty or anti-Myc (green) and anti-α/β tubulin (magenta) antibodies to label apical markers and microtubules, respectively. Images represent maximum-intensity projections of z-stack confocal sections, with inset zoom highlighting a single daughter apical complex. White arrowheads point daughter conoid. Scale bars are 1μM. **c-e**, Representative U-ExM images of apical markers in triple epitope-tagged APR8-mAID-HA_3_ strains following IAA depletion for 4h. Apical complex markers proximity was investigated in APR8-mAID-HA_3_ depleted parasites expressing CPH1-Myc-depleted tachyzoites in combination with either the PCR marker PCR1 (c), the upper-layer APR protein APR3 (d), or the middle-layer APR component APR2 (e). Intracellular tachyzoites were immunostained with anti-Myc (green) to label the conoid protein CPH1, anti-Ty (red) for Ty-tagged proteins, and anti-α/β-tubulin (white) for microtubules. Images represent maximum-intensity projections of z-stack confocal sections. White-contoured arrowheads point the mother apical complex (M: mother). Insets highlight developing daughter cells with white arrowheads indicating their apical complex (D: daughter). Scale bars are 2μM. **f,** Representative U-ExM images of RCC1–2-mAID-HA3 depleted parasites expressing APR8-Ty. Intracellular tachyzoites were stained with anti-HA (red), anti-Ty (green) and anti-α/β tubulin (white) antibodies to label RCC1–2, APR8 and microtubules, respectively. Images represent maximum-intensity projections of z-stack confocal sections. White-contoured arrowheads point the mother apical complex (M: mother). Insets highlight developing daughter cells (D: daughter). Scale bars are 5μM. Right: Quantification of APR8 signal at daughter apical complex upon RCC1–2 depletion. Presence of APR8 was quantified from approximately 30 daughter cells at early stage of endodyogeny and are presented as mean ± SD (n = 3 biological replicates). Statistical significance was determined by unpaired two-tailed Student’s t-test **g,** Schematic model illustrates a dividing tachyzoite with a zoomed inset on daughter apical complex organization under wild-type, RCC1–2-depleted and APR8-depleted conditions. RCC1–2 is positioned just beneath the bottom layer of the APR, whereas APR8 is a resident protein of the bottom layer APR. Both RCC1–2 and APR8 function as critical scaffolding proteins that govern distinct stages of DCS budding during endodyogeny. In RCC1–2-depleted tachyzoites, detached SPMTs are evident, demonstrating that RCC1–2 is essential for proper anchoring and stabilization of SPMTs to the APR.

We next quantified the proportion of tachyzoites expressing various apical complex markers after depleting APR8 for 6 hours with IAA. As expected at this time point, the depletion did not affect the apical structures of the mother (PCR, conoid and APR) (Fig. 7e; Supplementary Fig. 9a). For quantification in daughter cells, we measured signal presence in parasites exhibiting elongated SPMTs to avoid bias from markers not expressed at early stages of the budding process, such as CPH1 or even later markers KinA and APR7. The recruitment of all analyzed markers (CPH1, PCR1, APR3, APR2, KinA, and APR7) was affected to varying degrees (Fig. 8a, b). Approximately 20% and 30% of parasites failed to recruit the conoid (CPH1) and PCR (PCR1) markers, respectively. The defect reached 60% for the upper APR layer (APR3) and 70% for the middle APR layer (APR2), whereas the most pronounced disruption occurred in the bottom APR layer, with nearly all daughter cells lacked KinA and APR7. The presence of RCC1–2 in daughter cells was only slightly affected upon APR8 depletion (20%). The overall protein levels remained unchanged upon auxin treatment, demonstrating that the defects observed were not due to degradation of the inspected proteins (Supplementary Fig. 8c).

While the conoid marker CPH1 was discernible, the classical ring shape structure was never observed upon removal of APR8 (Fig. 8b-e; Supplementary Fig. 9d). Instead, the conoid adopted an abberant ‘arc’ shape, indicating that APR8 also plays a role in proper conoid maturation. TEM analysis further revealed nascent conoid in daughter cells, and that the IMC failed to align with the apical edge of the conoid, in contrast to wild-type parasites (Fig. 7e), illustrating improper assembly of daughter cell material. Notably, nascent conoid was still connected to centrioles via the SFA fiber in TEM analysis (Supplementary Fig. 9e), a result confirmed by tagging the SFA2 protein in APR8-mAID line and counting the number of parasites with SFA labeling by IFA (Supplementary Fig. 9f-h). This result indicates that division initiation and polarity establishment by the SFA fiber proceeded unperturbed in the absence of APR8. PCR1 staining appeared largely unaffected (Fig. 8b, c); however, its small size in daughter cells^31^ precluded definitive conclusions on its shape. PCR1 remained associated with one extremity of the arch formed by CPH1 in APR8-depleted parasites, as shown by costaining of the two proteins in APR8-mAID parasites (Fig. 8c) suggesting that APR8 is not associated in the recruitment of PCR1 and CPH1 to the PCRs and coinoid, respectively. In the absence of APR8, the upper and middle markers of APR—though sometimes still visible—lost their characteristic ring-shaped pattern (Fig. 8b). Both are visible close to conoid, but never formed a ring structure (Fig. 8d, e). Moreover, the daughter SPMTs sometimes radiate from the APR2 or APR3 signal. The transition of the arc to ring-like arrangement of RCC1–2 was also profoundly disrupted upon APR8 depletion (Fig. 8b). In contrast, tagging APR8 in RCC1–2-mAID strain (Supplementary Fig. 8b, c) showed that its normal localization and shape were largely preserved upon RCC1–2 depletion (Fig. 8f). This asymmetric dependency indicates that RCC1–2 functions downstream or independently of APR8 in apical complex assembly.

Together, these observations indicate a complete defect in multi-layered APR formation, despite recruitment of early markers APR3 and APR2 to nascent buds. This likely impairs subsequent recruitment of late APR markers KinA and APR7 and compromises proper SPMTs anchoring. In contrast, APR8 is dispensable for PCR and conoid initiation but essential for maturation and conoid assembly, establishing APR8 as a critical scaffold for coordinated daughter cell morphogenesis (Figure 8g).

## DISCUSSION

*T. gondii* tachyzoite endodyogeny is regulated at transcriptional and post-translational levels^18^. Recent methodological advances, such as U-ExM have shed light on DSC events^20,55^, highlighting the APR’s central role in DCS construction—yet no essential proteins for its formation had been identified until now. In this study, we describe two essential scaffolding proteins for DSC assembly: RCC1–2, which participates in anchoring SPMTs to the APR; and APR8, which ensures the correct assembly and maintenance of the multi-layered APR architecture. Both proteins are transiently expressed during parasite cell cycle: RCC1–2 persists until daughter cell maturation is complete, while APR8 disappears earlier, before daughter cell elongation. These dynamics indicate a scaffolding rather than permanent structural roles. Although both APR8 and RCC1–2 arrive early at the DSC construction site, their distinct localizations seem to be the determining factor for their specific role during daughter cell formation. APR8, a resident of the bottom layer APR, controls the overall assembly of the APR architecture: in its absence, most APR markers fail to associate with the nascent daughter bud or, when present, no longer display the typical ring-like organization. In APR8-depleted parasites, SPMTs are synthetized but are not always attached to a specific structure, reinforcing the role of the APR as the main anchor for the SPMT^56^, rather than a site for their nucleation as long proposed. RCC1–2, positioned beneath the basal APR layer, is required for robust SPMT attachment—a defect reminiscent of that observed in APR1/KinA double mutants^10^, both components of the basal APR. Interestingly, Leung et al. reported that the detachment of the SPMTs in the ΔKinAΔAPR1 parasites occur at later daughter development^10^. It is worth to mention that some bottom APR proteins, like RNG1^57^, APR1^55^ or APR7 (this study) are recruited late in the buding process. These observations suggest that the APR/SPMTs connection needs to be continously strenghtened through the recuitment of late components^55^. RCC1–2, which remains associated with the DSC until the completion of daughter maturation, RCC1–2, which remains associated with the DSC until the completion of daughter maturation, is an ideal scaffolding protein to sustain this function throughout DSC assembly. In contrast to APR8, RCC1–2 depletion does not grossly perturb APR architecture, as shown by APR-layer markers and cryo-ET, with APR7 being the only marker that loses its typical ring-like distribution. APR7 spans the interface between the APR and the apical ends of SPMTs, although its precise position remains unresolved. Consistently, RCC1–2 sits just beneath APR7^16^, and cryo-ET of RCC1–2-depleted parasites reveals SPMTs detachment and shortened interspersed pillars. Together, these observations demonstrate that RCC1–2 is required for proper interspersed pillars assembly and establish these newly described elements as critical anchors for SPMTs at the APR. Notably, the IMC remains correctly attached to the APR in RCC1–2-depleted parasites, indicating that APR-IMC linkers are intact. Since interspersed pillars have been proposed to occupy the expanded region below the amorphous AAD ring that links the APR to the IMC, our data point to a narrow function for RCC1–2 in building the interspersed pillars themselves, and that other scafolding proteins likely contribute to connect the IMC to the AAD ring. As a transient DCS component, RCC1–2 is well placed to recruit yet-unknown pillar constituents.

In addition to defective SPMTs anchorage, RCC1–2 depletion causes interruptions in the IMC, which could arise from mechanical stress during cytoskeletal elongation when SPMTs detach from the APR. This detachment may also contribute to altered basal complex morphology observed. However, because apical and basal poles form concomitantly early during DCS assembly ^21,24^, RCC1–2 might also directly influence IMC organization, including plate sutures and basal pole development, independently of its role in SPMT anchoring.

In this study, we demonstrate that the four APR layers assemble concomitantly during nascent APR formation—with their distinct localizations already established prior to conoid assembly—suggesting that this hierarchy is established at the onset of their insertion into the nascent APR. We further detect PCR1, a PCR marker^14^, at the same early DCS stage, and similar early recruitment is seen for Centrin2, which also localizes to the PCR^55^. Thus, APR and PCR jointly pioneer apical pole construction ahead of cytoskeletal structures such as the conoid or ICMTs. To date, SFA fiber nucleation is the only event known to precede nascent APR appearance^4,25^. The absence of perturbation of the SFA2 marker in absence of APR8 suggests that the SFA fiber nucleation happens upstream and independently from of the nascent APR formation. In fact, the function of the SFA fiber precedes that of γ-tubulin as shown in an auxin-inducible mutant of SFA2^20^. SFA fiber has been hypothesized to act as a spatial organizer of the DCS formation site^25^, but its function needs to be further investigated to understand it role in APR formation.

In parallel, an independent study identified APR8 (named ASAF1) as a crucial factor for daughter cell formation^58^. In our study, we precisely localized this protein to the APR basal layer and adopted the APR-based nomenclature by naming it APR8. Our work confirms most of their findings but also reveals differences attributable to the depletion strategies used. Here, we employed a fast-acting auxin-inducible degron that depletes APR8 within tens of minutes, enabling phenotypic analysis after 4–6 h of depletion, before secondary defects accumulate. By contrast, Li et al.^58^ used the inducible Cre system^59^ that induces genetic excision of the target sequence in a much slower kinetic (24 to 48 hours). The fast degradation of APR8 in our system allowed an evaluation of the phenotype after 4–6 hours of depletion, before the accumulation of indirect phenotypes, and a close inspection of the the apical complex structures formation in absence of APR8. In our system, PCR, conoid, and APR markers are clearly recruited near duplicated centrioles, yet none achieve their normal morphology. The conoid consistently appears as an immature arc reminiscent of the primordial conoid seen by U-ExM^55^, indicating a maturation rather than a recruitment defect. PCR1 remains associated with the conoid, typically at one extremity, but its small size precludes definitive structural assessment. By contrast, APR2 (middle layer) and APR3 (upper layer) are often undetectable at the apical pole and, when present, never form rings. Likewise, RCC1–2—marking the deepest layer/interspersed pillars—is recruited but fails to assemble into a continuous ring, and the late basal APR markers APR7 and KinA are not detected near the conoid. Together, these data indicate a profound failure of APR assembly upon APR8 loss, which compromises downstream events such as anchoring of newly polymerized SPMTs and the IMC, as also seen by TEM. Although PCRs and the conoid still initiate without APR8, the abnormal conoid shape underscores that its maturation is coupled to global APR integrity. Recent structural and molecular work shows that the conoid-APR connection relies on tethering elements formed by RNG2, which bridges the conoid to the APR in mature parasite^42,60,61^. Notably, RNG2 depletion does not block conoid formation or maturation but causes its detachment from the apical complex in mature parasite, suggesting that other APR proteins are crucial for stabilization and maturation of conoid during endodyogeny. The APR ring may also template the conoid’s transition into a fully cylindrical structure. The fact that APR8 is dispensable for initial PCR or conoid formation supports the hypothesis that the PCR—which precedes conoid assembly—might act as an organizing center for conoid biogenesis. The orientation of conoid fiber minus ends toward the PCR, with plus ends near the APR, further aligns with a model in which the PCR organizes conoid assembly. To date, no mutants specifically defective in PCR biogenesis have been identified to test this hypothesis. Known mutants such as GCP, PCR4, and PCR5^31,58^, are crucial for maintaining a mature PCR but dispensable for its initial formation, suggesting that additional, as-yet-unknown factors drive PCR initiation. PCR7, a transiently expressed PCR component during DCS formation^14^, would be an attractive candidate, but its non-essentiality argues against a primary scaffolding role. Similarly, depletion of ERK7 induces a complete loss of the PCR, conoid, and APR, but this effect is observed only in mature parasites. The core scaffolding proteins that directly organize PCR and conoid architecture therefore remain to be identified^46,47^.

Both scaffolding proteins, APR8 and RCC1–2, are specific to coccidian parasites—a subclass of apicomplexans (including *Eimeria* spp.) characterized by an elaborate conoid. This coccidian specificity might reflect parasite-specific features of the APR and its considerable variability among apicomplexans and across developmental stages. In *Toxoplasma*, the APR appears as three distinct density layers from which 22 evenly spaced SPMTs radiate. In *Plasmodium*, APR structure varies markedly by stage^62,63^: merozoites and sporozoites have a single ring, with 2–3 asymmetrically arranged SPMTs in merozoite, while sporozoites feature 13 closely spaced SPMTs plus one opposing microtubule at tilted angles. Ookinetes exhibit the most complex “apical collar” with two concentric layers and basal tentacles supporting ~50–60 staggered SPMTs. These divergent architectures illustrate how stage- and species-specific APR designs tune cell shape and mechanics.

Overall, our study pionnered the understanding of the molecular players of the initiation of the apical complex construction, specifically the APR and connecting materials with SPMTs of *Toxoplasma gondii*. By defining these early assembly factors and their spatial organization, our work connects the molecular blueprint of APR-SPMTs coupling and links it to the apical complex’s essential roles gliding motility, host cell invasion, and egress.

## Materials & Methods

### *Toxoplasma* culture conditions - Antibodies used

*T. gondii* RH tachyzoites (Type I strain) were routinely grown in Human Foreskin Fibroblasts (HFFs, ATCC CRL 1634) in Dulbecco’s Modified Eagle Medium (DMEM, Gibco) supplemented with 5% fetal bovine serum (FBS, Biosera), 2mM glutamine (Gibco) and 1% penicillin/streptomycin (Gibco) at 37°C + 5% CO_2_. Transgenic lines were generated using either the RHΔKu80TATi strain, which expresseds the TATi transactivator for the TetOff system^64^, or the RHΔKu80TIR1 strain, which expresses the Tir‐1 receptor for the auxin inducible degron system^65^ (miniAID). Mycophenolic acid (25 μg/mL) and xanthine (50 μg/mL) were added to the culture media to positively select transformants carrying the hypoxanthine–xanthine–guanine phosphoribosyl transferase (HXGPRT) drug resistance cassette. For conditional knockdown, 0.5 mM auxin (indole‐3‐acetic acid, referred to as IAA (Sigma-Aldrich)) was added to the medium.

Antibodies used and their dilution are listed in Supplementary Table 4.

### Generation of *Toxoplasma* tagged and knockdown strains

All parasite strains used in this study were generated by CRISPR/Cas9-mediated homology-directed repair. For C-terminal epitope tagging, 40 μg of the pU6-Cas9-YFP carrying the sgRNA targeting the 3′ untranslated region (3′UTR) of the gene of interest (GOI) was co-transfected with a PCR product containing the corresponding homology regions and the desired tagging cassette (mAID-HA-HXGPRT, HA-TurboID-DHFR, Ty-DHFR, or Myc-DHFR). The mAID-HA-HXGPRT cassette was amplified from the plasmid pTUB8YFP‐mAID‐3^65^. The pU6-sgRNA plasmids were constructed by annealing and cloning the sgRNA primers into the pU6-Cas9-YFP plasmid using the BsaI restriction sites. The list of all tagged lines, primers and RNA guides (gRNAs) used are listed in Supplementary Table 4.

For all constructs, freshly egressed *T. gondii* tachyzoites (20 × 10^6^) were electroporated with approximately 15 μg of the pU6-Cas9-YFP plasmid and 100 μL of PCR-amplified homologous recombination (HR) template using an Electro Cell Manipulator 630 (BTX) with the following settings: 2.02 kV, 50 Ω, and 25 μF^66^. Transfected parasites were allowed to infect HFFs monolayers. After transfection, positive transformants were recovered by drug selection for transformants carrying the hypoxanthine–xanthine–guanine phosphoribosyl transferase (HXGPRT) drug resistance cassette or by fluorescence-activated cell sorting (FACS) based on Cas9-YFP fluorescence for C-terminal tagging with HA-Turbo, Myc or Ty epiopes. Clones were isolated by limiting dilution and screened by PCR for correct integration with Phire^™^ Tissue Direct PCR master mix (Thermo Scientific) protocol, as previously described^67^. The primers used to validate the integration are listed in Supplementary Table 4.

### *Toxoplasma* invasion assay

HFF cells grown on coverslips were infected with freshly egressed *T. gondii* tachyzoites (5. 10^6^/coverslips) pre-treated for 24h ± IAA. Parasites were incubated on ice for 20 min to synchronize invasion prior to being placed in a 38.5°C water bath for 5 min to allow invasion. Infected cells were fixed in 4% paraformaldehyde (PFA) (w/v) in Hank’s Buffer Salt Solution (HBSS; Gibco) for 20 min at room temperature. To distinguish intracellular from extracellular parasites, immunofluorescence assays were performed using a dual-staining antibody as previously described^68^. Non-permeabilized extracellular parasites were incubated first with 10% FBS/HBSS blocking solution and then stained using mouse mAbs T41E5 anti‐SAG1 antibodies^69^ (1:2,000^66^) in 2% FBS/HBSS at 37°C. To visualize intracellular parasites, samples were then permeabilized with 0.1% saponin in PBS for 15 min, incubated in blocking solution for 1 hour and stained using rabbit anti-GAP45 antibodies (1:9,000^70^) with 2% FBS in Phosphate Buffered Saline (PBS). After three washings with PBS, the samples were incubated with AlexaFluor594 goat anti‐mouse (1:4,000) and AlexaFluor488 goat anti‐rabbit (1:10,000) secondary antibodies (Invitrogen). Extracellular and intracellular parasites were scored in 20 fields per coverslips in triplicates with a Leica Thunder microscope, with a 100× oil objective NA = 1.4, equipped with the sCMOS 4.2MP camera, using Leica Application Suite X (LAS X) software (Leica Biosystems). The values were normalized to that of the control line (RHΔKU80 Tir1).

### *Toxoplasma* plaque assay

HFF cells grown in 24-well plates were infected with 7000 of freshly egressed tachyzoites per well. After a serial dilution of *T. gondii* tachyzoites, parasites were allowed to grow for 7 days with or without IAA at 37°C + 5% CO_2_. HFF cells were then fixed with 4% PFA/PBS for 20 min, washed once with PBS prior to be stained with 1% crystal violet (Sigma). Image of the lysis plaques were acquired with Olympus MVX10 macro zoom microscope, equipped with a Zeiss MRM2 Camera. Lysis plaque area was measured using ImageJ software. Graphs are presented as mean values from three independent experiments, including three technical replicates each.

### *Toxoplasma* replication assay

HFF cells grown on coverslips were infected with 2×10^5^ of freshly egressed *T. gondii* tachyzoites for 2 hours to allow invasion. HFFs cells were then washed five times with HBSS to remove remaining extracellular parasites. Parasites were then allowed to grow with or without IAA at 37°C for 16 hours. Parasites were fixed with 4% PFA/PBS for 20 min, permeabilized with 0.1% Triton X-100 and incubated with 5% BSA/PBS blocking solution. Immunofluorescence assay was performed with rabbit anti-GAP45^70^ (1:9,000) and AlexaFluor488 goat anti-rabbit (1:4,000; Invitrogen) antibodies diluted in 2% BSA/PBS. Nuclei were stained with 16 μM Hoechst before mounting the coverslips onto microscope slides using Immunomount (Calbiochem). The number of vacuoles containing either 1, 2, 4, or 8 nuclei per vacuole were scored with a Leica Thunder microscope, with a 100× oil objective NA = 1.4, equipped with the sCMOS 4.2MP camera, using Leica Application Suite X (LAS X) software (Leica Biosystems) and expressed as a percentage. A total of 200 vacuoles/coverslips were scored for each condition. Graphs are presented as mean values from three independent experiments, including three technical replicates each.

### *Toxoplasma* egress assay

HFF cells grown on coverslips were infected with 1×10^5^ of freshly egressed *T. gondii* tachyzoites following 30h of infection in presence or absence of IAA. To stimulate egress, infected cells were incubated with calcium ionophore A23187 (3 μM) or dimethyl sulfoxide (DMSO, control) for 8 min at 37°C w and then fixed with 4% PFA/PBS for 20min. Immunofluorescence assay was performed using rabbit anti‐GAP45^70^ (1:9,000) and mouse anti-GRA3 hybridoma^71^ (1:100) followed by incubation with AlexaFluor594 goat anti‐rabbit (1:4,000) and AlexaFluor488 goat anti‐mouse (1:4,000) antibodies (Invitrogen). Nuclei were stained with 16 μM Hoechst before mounting the coverslips onto microscope slides using Immunomount (Calbiochem). Egress events were analyzed by assessing the presence of GRA3 proteins in intact and ruptured parasitophorous vacuoles stained with GAP45. 200 vacuoles were scored for each condition. Graphs are presented as mean values from three independent experiments, including three technical replicates each.

### Western blotting of *Toxoplasma* proteins

For western blotting of infected HFF monolayers, parasites were cultured 30h prior to be harvested by scraping and syringing out 3 times with a 25-gauge needle. Parasites were pelleted by centrifugation at 800 × *g* for 5 min, washed once in PBS, pelleted again and resuspended in Laemmli buffer 4X supplemented with 40 mM dithiothreitol (DTT; Sigma). Proteins extracts were separated on 10% Novex NuPAGE Gels (Invitrogen) and transferred to 0.45 μm PVDF (Immobilon^®^‐P, Millipore) membranes. Blots were blocked either with 5% dried milk or 3% BSA in TNT (15 mM Tris, 140 mM NaCl, and 0.05% Tween 20, pH 8) and incubated with antibodies diluted in blocking solution. Detection was performed using Clarity Max^™^ Western ECL (Bio‐Rad) and imaged with a Chemidoc System (Bio‐Rad).

### Sodium deoxycholate extraction

Freshly egressed parasites pre-treated for 24h ± IAA were settled onto Poly-L-Lysine coated coverslips by centrifugation at 400 × *g* for 1 min. Subsequently, parasites were treated with DMSO (as control) or 10 mM of sodium deoxycholate (DOC; ThermoFisher) for 20 min at room temperature. Parasites were then fixed with ice-cold methanol for 8 minutes prior to immunostaining using mouse anti-acetylated αtubulin (Santa Cruz Biotechnology, sc-23950) and rabbit anti-HA (Abcam) antibodies followed by incubation with AlexaFluor488 goat anti-mouse (1:4,000) and AlexaFluor594 goat anti‐rabbit (1:4,000) antibodies (Invitrogen).

### Biotinylation by TurboID

RCC1–2^HA3-TURBOID^ parasites were cultured in HFFs monolayers for 28 hours in normal media. After a wash with biotin-free medium, parasites were then grown in biotin-free DMEM (as control) or biotin-free DMEM supplemented with 150 mM D-biotin (Sigma-Aldrich) for 2 additional hours. Intracellular parasites were harvested by scraping and syringing out 3 times with a 25-gauge needle, washed in PBS and then lysed with a cytoskeletal lysis buffer 1 (50 mM Tris [pH7.5], 150 mM NaCl, 0.1% SDS, 0.5% sodium deoxycholate, 1% NP-40, Complete Protease Inhibitor Cocktail (Roche)) for 30 min on ice. Lysates were centrifuged for 15 min at 14,000 × *g* at 4°C. Following centrifugation, the pellet corresponding to the cytoskeletal and IMC fraction was lysed using lysis buffer 2 (50 mM Tris [pH7.5], 150 mM NaCl, 1% SDS, 1% Triton X-100, Complete Protease Inhibitor Cocktail (Roche)) for 30 min and then incubated with Steptavidin magnetic beads (ThermoFisher Scientific) overnight at 4°C under agitation. Beads were collected and washed five times with wash buffer (50 mM Tris [pH7.5], 150 mM NaCl, 0.1% SDS, 1% Triton X-100, Complete Protease Inhibitor Cocktail (Roche)) prior to be eluted in Laemmli buffer 4X. Supernatants from the eluates were loaded onto a 10% acrylamide SDS-PAGE gel, transferred to 0.45 μm PVDF (Immobilon^®^‐P, Millipore) membranes. Blots were blocked with 3% BSA in TNT and incubated with horseradish peroxidase (HRP)-conjugated Streptavidin (1:5,000; Sigma-Aldrich) antibodies diluted in blocking solution. Detection was performed using Clarity Max^™^ Western ECL (Bio‐Rad) and imaged with a Chemidoc System (Bio‐Rad).

For IFA, infected monolayers were incubated for 2 h with biotin prior to fixation with 4% PFA for 20 min. Coverslips were then processed according to the standard immunofluorescence staining protocol described for invasion and replication assays.

For mass spectrometry analysis, electrophoresis was stopped once the samples had migrated 1–2 cm into the gel. Proteins were stained with colloidal blue (Invitrogen) prior to be analyzed by mass spectrometry (MSPP platform – INRAE Montpellier).

### Mass spectrometry analysis of *Toxoplasma* proteins

#### Sample preparation

Gel bands were manually cut and and re-cut into small pieces (2mm2) that were transferred to 2 mL low binding tubes (Thermo Scientifics). Bands were first washed with 500 μl of water and then 500 μl of 25 mM NH4HCO3. Destaining was performed twice in the presence of 500 μl of 50% acetonitrile (ACN) in 25 mM NH_4_HCO_3_. Gel bands were dehydrated twice by 500 μl of 100% acetonitrile, and finally dried at room temperature. Destaining is followed by the reduction of disulfide bridges with 500 μl of 10 mM DTT at 56°C for 45 min, then supernatant was removed and cysteins were alkylated with 500 μl of 55 mM iodoacetamide during 30 min on a vortex in dark. Gel bands were washed two times with 500 μl of 50% ACN in 25 mM NH_4_HCO_3_, then dehydrated by 500 μl of 100% ACN, and finally dried at room temperature. 30 microliters of a trypsin solution (Sequencing Grade Modified Trypsin, Promega, Madison, USA), at a concentration of 0.0125 μg/μl in 25 mM NH_4_HCO_3_, was added to every gel region, and sufficient carbonate buffer (25 mM NH_4_HCO_3_) was added to cover the gels and were kept for 10 min on ice. A volume of carbonate buffer was added once more to cover the gels before the samples were kept another 10 min at room temperature. Digestion was performed overnight at 37°C then peptides were extracted by addition 100 μl formic acid (FA) 2 %. Digests were first voretexed for 10 minutes and supernatants were transferred into 2 mL low binding tubes. Gel bands were extracted twice by addition of 100μL of 80 % ACN. After solvent evaporation in a vacuum centrifuge, peptides were resuspended in 10 μl 2% FA. Then purified with a micro tip C18 (Zip-Tip- C18 Millipore Corpration Billerica MA, USA). Peptides were eluted with a solution containing 2% FA (v/v) and 80% ACN (v/v) and dried in a vacuum centrifuge until total evaporation. Peptides were resuspended in 8 μl 2% FA before LC-MS/MS analysis

#### Mass-spectrometry analysis with the Thermofisher scientific Exploris-240

The LC-MS/MS experiments were performed using an Ultimate 3000 RSLC nano system (Thermo Fisher Scientific Inc, Waltham, MA, USA) interfaced online with a nano easy ion source and an Exploris 240 Orbitrap mass spectrometer (Thermo Fisher Scientific Inc, Waltham, MA, USA). The samples were analyzed in Data Dependent Acquisition (DDA). 6.5 μl of peptides were first loaded onto a pre-column (Thermo Scientific PepMap 100 C18, 5 μm particle size, 100 Å pore size, 300 μm i.d. × 5 mm length) from the Ultimate 3000 autosampler with 0.05% TFA in water at a flow rate of 10 μL/min. The peptides were separated by reverse-phase column (Thermo Scientific PepMap C18, 2 *μ*m particule size, 100 Å pore size, 75 *μ*m i.d. 50 cm length)/ at a flow rate of 300 nl/min. After 3 min loading period, the column valve was switched to allow elution of peptides from the pre-column onto the analytical column. Loading buffer (solvent A) was 0.1% FA in water and elution buffer (solvent B) was 0.1% FA in 80% acetonitrile. The linear gradient employed was 2–25% of solvent B in 103 min, then 25–40% of solvent B from 103 to 123 min, finally 40–90% of solvent B from 123 to 125 min. The total run time was 150 min including a high organic wash step and re-equilibration step. Peptides were transferred to the gaseous phase with positive ion electrospray ionization at 1.9 kV. In DDA the top 15 precursors were acquired between 350 and 1200 m/z with a 2 Th (Thomson) selection window, dynamic exclusion of 40 s, normalized collision energy (NCE) of 30 and resolutions of 120 000 for MS and 15 000 for MS2. Spectrum were recorded with Xcalibur software (4.7.69.37) (Thermo Fisher Scientific).

#### Identification and quantification

The .raw files were analysed with MaxQuant version 2.0.3 using default settings. The minimal peptide length was set to 6. The criteria “Trypsin/P” (which means C-terminus peptides of “K/R” unless followed by “P”: “K/R” followed by “P” cannot be a cleavage site) was chosen as the digestion enzyme. Carbamidomethylation of cysteine was selected as a fixed modification and as variable modifications: N-terminal acetylation of protein; oxidation of methionine; N-terminal-pyroglutamylation of glutamine and glutamate. Up to two missed cleavages were allowed. The mass tolerance for the precursor was 20 and 4.5 ppm for the first and the main searches, respectively, and for the fragment ions it was 20 ppm.

The files were searched against *Toxoplasma gondii* (March 2020 -https://www.uniprot.org/proteomes/UP000005641-8450 entries) and the Maxquant contaminant database. Identified proteins were filtered according to the following criteria: at least two different trypsin peptides with at least one unique peptide, an E value below 0.01 and a protein E value smaller than 0.01 were required. Using the above criteria, the rate of false peptide sequence assignment and false protein identification were lower than 1%. Proteins were quantified by the label-free method with MaxQuant software using unique and razor peptide intensities ^72^. Statistical analyses were carried out using RStudio package software (t-test with p < 0.05 or 0.01 with or without Benjamini correction) to identify the significant differences in the protein abundance. Proteins were retained if they were quantified in at least two of the three replicates in at least one experiment.

Then the protein intensity ratio was calculated (protein intensity in test condition / protein intensity in control) to determine if has up-regulated or down-regulated. In some cases, certain proteins may be absent in one of the experimental conditions. To confirm the absence of a protein, we required that it be undetected in all three replicates of the given condition, while being consistently detected in all three replicates of the other condition. In such cases, protein presence or absence was determined based on the detection of unique peptides only.

### Transmission electron microscopy

HFF cells grown on Nunc Lab-tek chambers with Permanox bottom were infected with freshly egressed *T. gondii* tachyzoites following 24h of infection in presence or absence of IAA. The culture medium was replaced by the same volume of fixative (2.5% glutaraldehyde, in 0,1M sodium cacodylate pH7.4) and fixed for 2 hours at RT, then stored at 4°C in the fixative until further processing. The fixed cells were then washed twice in cacodylate buffer 0,1M to remove the glutaraldehyde. For scanning electron microscopy (SEM), embedding in Epon was performed using a modified version of the protocol of Hua et al.^73^ All the following steps were performed using a Pelco Biowave Pro+ tissue processor (Ted Pella). The details of the program are indicated in Supplementary Table 5. Briefly, the cells were first fixed with a double osmium staining procedure as follows : **1)** 1% OsO_4_, 5 mM CaCl_2_, in cacodylate buffer 0,1 M pH 7.4; **2)** Cacodylate buffer washes; **3)** *In situ* reduction with potassium ferricyanide 1.5 %, 5mM CaCl_2_ in cacodylate buffer; **4)** distilled water washes; **5)** 1% thiocarbohydrazide (TCH) in water **6)** water washes **7)** 1% OsO_4_ in water; **8)** distilled water washes. The samples were then incubated in 2% uranyl acetate at 4°C overnight. The next day, the samples in uranyl acetate were heated 10 min at 37°C and processed with the Biowave (without washing). After processing, the samples were washed with water, incubated in lead aspartate, washed with water, and then dehydrated in growing concentrations of acetonitrile. Finally, they were progressively impregnated in EPON Hard^+^ resin directly into the Labtek. The resin was then polymerized at 60°C in an oven for 48 hours. TCH and lead aspartate were prepared extemporaneously as described in Deerinck et al.^74^. For transmission electron microscopy (TEM), samples were simply stained with osmium (as above, step 1), incubated overnight in 2% uranyl acetate, and the dehydrated and epon embedded as for SEM.

Thin serial sections of 70 nm were prepared with a Leica UCT ultramicrotome using a 35° Jumbo ultra Diatome diamond knife. For SEM, sections were collected on silicon wafers that has been hydrophilized by glow discharge using a Safematic CCU-010 carbon coater equipped with an etching unit (30 seconds of plasma treatment at 8.10^−1^ mbar, 15mA, in air). For TEM, sections were collected on 100 mesh copper grids coated with collodion and post-stained with uranyl acetate and lead citrate.

SEM images were acquired at the MRI-EM4Bio facility using a Zeiss Gemini 360 scanning electron microscope using the Volutome-BSD detector. The wafers were mounted on sample stubs using double-sided adhesive copper tape. Automated acquisitions were performed using Atlas 5 software for array tomography using the following parameters: voltage: 1.5 kV; aperture 30 μm; working distance 4 mm, dwell time 3.2μsec. Autofocus was applied once per tile and autostigmatism once per mosaic. Serial section series were aligned with Atlas and manually segmented using DragonFly 3D world software.

TEM images were acquired at the MEA facility on Jeol 1010 TEM equipped with a Quemsa Emsis camera at 80kv.

### Preparation of *Toxoplasma gondii* tachyzoites for cryo-electron tomography

HFF cells were maintained in T25 flasks in Dulbecco’s Modified Eagle Medium (DMEM; Gibco) supplemented with 5% fetal calf serum (FCS) and 2 mM glutamine. Two confluent flasks were infected with RCC1–2-mAID-HA *T. gondii* parasites strain. At 38 hours post-infection, when parasites were fully intracellular, cultures were washed five times to remove extracellular parasites and subsequently treated with or without indole-3-acetic acid (IAA). After 10 hours of the treatment, freshly egressed tachyzoites were collected and resuspended in phosphate-buffered saline (PBS) containing 10 nm gold fiducials. Approximately 4 × 10^6^ parasites in a total volume of 4 mL were applied to EM grids and plunge-frozen into a liquid ethane/propane mixture using an EM GP2 automated plunger (Leica Microsystems, Wetzlar, Germany) following 4–6 sec of front blotting.

### Cryo-electron tomography data collection and processing

Cryo-electron tomography was performed on a Thermo Fisher Scientific Titan Krios G3i 300 keV field-emission cryo-transmission electron microscope equipped with an energy filter. Dose-fractionated images were acquired using SerialEM software on a K3 direct electron detector (Gatan) operated in electron-counting mode. Movie frames were motion-corrected using the Alignframes function in IMOD. Tilt series were collected using a dose-symmetric acquisition scheme over an angular range of −60° to +60° with 2° increments at a nominal magnification of 33,000×, corresponding to a calibrated pixel size of 2.67 Å, and at a defocus of approximately −5 μm. The cumulative electron dose per tilt series was approximately 120 e^−^ Å^−2^. Tilt series alignment was performed through fiducial-based tracking in Etomo (IMOD version 4.11.25) using the 10 nm colloidal gold bead as fiducial markers. The resulting alignment parameters were subsequently imported into Warp (Version 2.0.0) for CTF correction and reconstruction. Reconstructed tomograms were binned by a factor of 3.7, yielding a final voxel size of 10 Å. Tomogram quality was further improved through denoising with IsoNet2 (v2.0.0-beta). The resulting denoised tomograms were used for segmentation in DragonFly (Version 2025.1).

### Conoid extrusion assay

Tachyzoites pre-treated for 24h ± IAA were lysed by 3 passages through a 25-gauge needle, then then added to poly-D-Lysine-coated coverslips pre-heated at 37°C. The plate was centrifuged at 400 × *g* for 1 min to attach the parasites to the coverslips. Attached parasites were incubated at 37°C for 8 min in pre-heated HEPES solution (274 mM NaCl, 10 mM KCl, 2 mM Na_2_HPO_4_, 11 mM glucose, and 42 mM HEPES, pH 7.05) supplemented with 5 mM CaCl_2_ and 5 μM A23187 calcium ionophore to induce the extrusion of conoid. As control, parasites incubated in HEPES solution without A23187 were used. Standard U-ExM protocol was then followed using anti-HA and anti-α/β tubulin primary antibodies.

### Ultrastructure expansion microscopy (U‐ExM) of *Toxoplasma* tachyzoites

Intracellular vacuoles in HFFs, or syringe-lysed tachyzoites seeded onto poly-D-lysine (Gibco) coated coverslips , were transferred in a protein cross-linking solution (2% acrylamide/0.7% formaldehyde in PBS) for 3 h at 37°C. Cells were then embedded in a gelation solution (19% sodium acrylate/10% acrylamide/0.1% N,N′-methylenbisacrylamide/10% TEMED/10% APS in PBS) on ice, prior to gelation for 1 h at 37°C. Gels were then incubated in denaturation buffer (200 mM SDS, 200 mM NaCl, 50 mM Tris pH 9) for 15 min under agitation to facilitate their detachment from coverslips. Gels were placed in Eppendorf tubes filled with denaturation buffer and incubated at 95 °C for 90 min. Gels were then expanded overnight in ddH_2_O at room temperature, then shrank by two 15-min washes in PBS prior to antibody incubation. The gel slices were incubated with primary antibodies diluted in 2% BSA/PBS at 37°C for 3 h. Gels were then washed three times for 10 min in PBS containing 0.1% Tween 20, followed by incubation with secondary antibodies at 37°C for 3 h. Gels were washed three times for 10 min in PBS containing 0.1% Tween 20 before the final expansion step, performed by incubating the gels in ddH_2_O overnight. Gel slices were then placed onto poly-D-lysine-coated 24-mm coverslips and imaged with a Zeiss LSM880 confocal microscope equipped with Airyscan detector, with a 63×1.4 NA oil objective, using Zen Black software (Zeiss, Intelligent Imaging Innovations). Z‐stacks were denoised, adjusted in brightness and contrast, colored, and processed to obtain maximum intensity projections using the Fiji software. Three-dimensional reconstructions of intracellular and extracellular parasites were generated using Imaris (version 9.9, Bitplane/Oxford Instruments). After confocal image stacks import, voxel size and channel parameters were set according to the acquisition metadata. Structures of interest (e.g., tubulin-based cytoskeleton, basal complex and RCC1–2 protein) were segmented using the Surface module based on their respective marker channel. For the subpellicular microtubules, the threshold level was determined from control images to include continuous SPMTs while excluding diffuse background. The resulting surfaces were visually inspected in 3D view and, where necessary, edited manually to remove spots outside the parasite.

## Supplementary Material

Supplement 1

Supplement 2

Supplement 3

Supplement 4

Supplement 5

Supplement 6

Supplement 7

Supplement 8

Supplement 9

Supplement 10

Supplement 11

## Data Availability

The mass spectrometry proteomics data have been deposited to the ProteomeXchange Consortium via the PRIDE ^75^ partner repository with the dataset identifier PXD074368.
