# Supplementary material for: Two anchoring proteins control daughter apical complex assembly in Toxoplasma gondii
Source: bioRxiv. 2026 Feb 14:2026.02.13.705759. Preprint. [Version 1] doi: 10.64898/2026.02.13.705759 (PMC12918799; doi:10.64898/2026.02.13.705759)
Supplement: Supplement 11 [file NIHPP2026.02.13.705759v1-supplement-11.pdf]

Supplementary Fig. 1

a

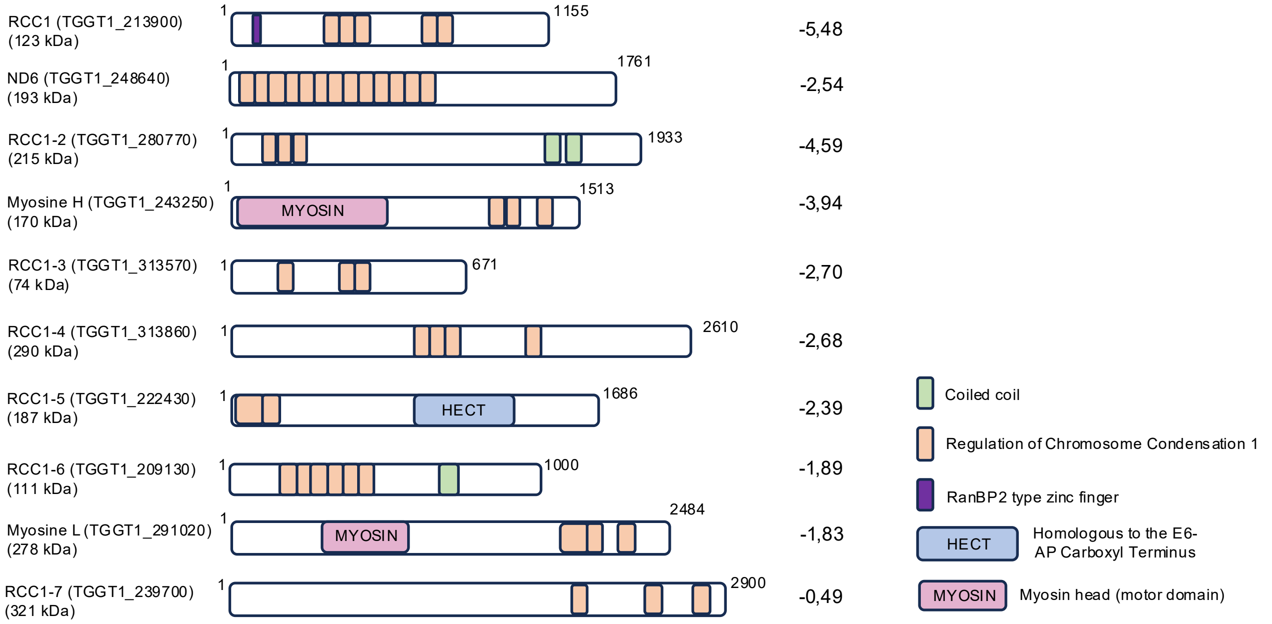

b

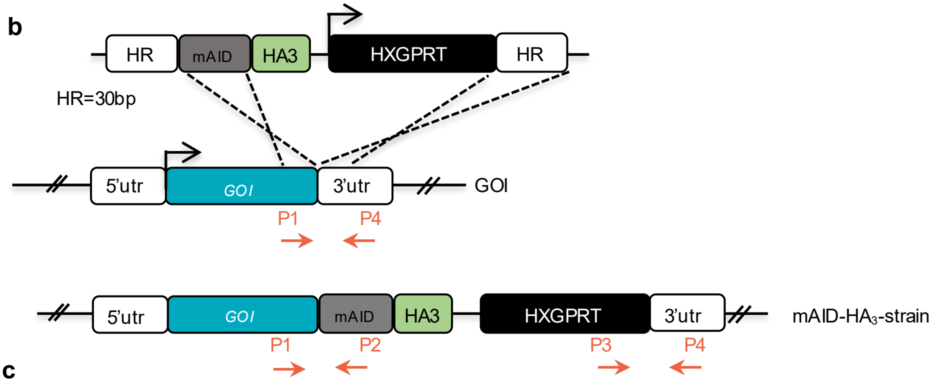

c

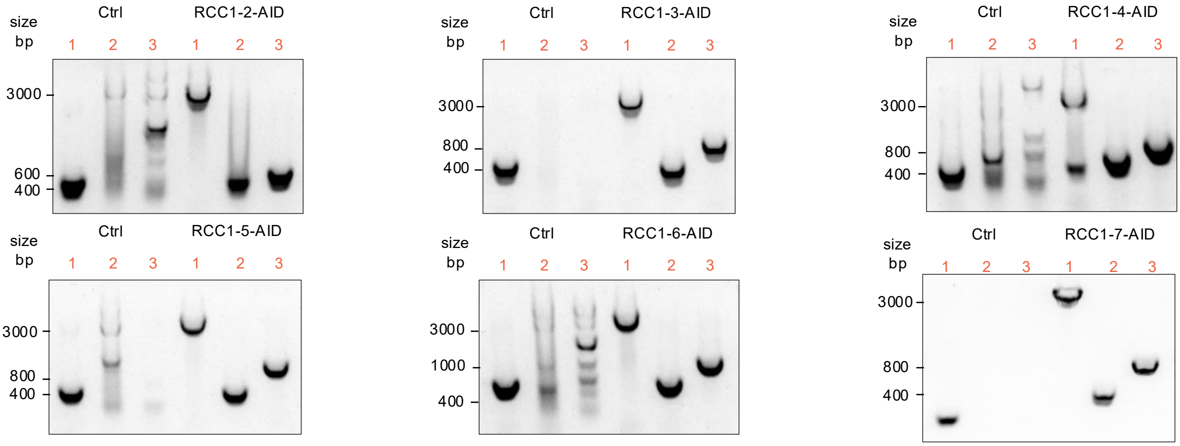

	RCC1-2	RCC1-3	RCC1-4	RCC1-5	RCC1-6	RCC1-7
1: P1+P4	2865bp	3042bp	3035bp	3000bp	3233bp	2908bp
2 : P1+P2	348bp	384bp	523bp	375bp	523bp	362bp
3 : P3+P4	763bp	815bp	758bp	870bp	956bp	792bp
Ctrl P1+P4	291bp	484bp	340bp	395bp	520bp	192bp

bioRxiv preprint doi: https://doi.org/10.64898/2026.02.13.705759; this version posted February 14, 2026. The copyright holder for this preprint (which was not certified by peer review) is the author/funder, who has granted bioRxiv a license to display the preprint in perpetuity. It is made available under aCC-BY 4.0 International license.

Supplementary Figure 1. Characterization of RCC1-repeats containing proteins in *T. gondii.* **a,** Schematic representation of the RCC1-repeats containing proteins expressed during S/M phase and their INTERPRO-annotated functional domains[51]. RCC1: regulator of chromosome condensation 1-like domains (RLDs) (shown in orange). HECT : Homologous to the E6-AP Carboxyl Terminus (shown in blue), a catalytic domain founds in E3 ubiquitin-protein ligases[62]. Coiled-coil domain[63] (shown in green) . RanBP2: RanBP2 type zinc finger domain (shown in purple), a prevalent domain founds in nucleoporins and splicing factors[64]. MYOSIN: myosin motor domain (shown in pink), a conserved motor head domain that binds ATP and actin filaments to generate force and movement[65]. **b,** Illustration of the cloning strategy employed to obtain the -mAID-HA$_3$ strain using CRISPR/Cas9 (top panel). Orange arrows depicted the designed primers used to validate the successful integration at the endogenous locus. **C,** Validation of successful integration of the mAID-HA$_3$ cassette into *TgRCC1-2* (*TGGT1_280770)*, *TgRCC1-3* (*TGGT1_313570)*, *TgRCC1-4 (TGGT1_313860)*, *TgRCC1-5 (TGGT1_222430), TgRCC1-6 (TGGT1_209130)*, *TgRCC1-7 (TGGT1_239700)* locus by PCR. The parental strain Tir1 served as a negative control (Ctrl). Table lists expected PCR product sizes for each primer pair.

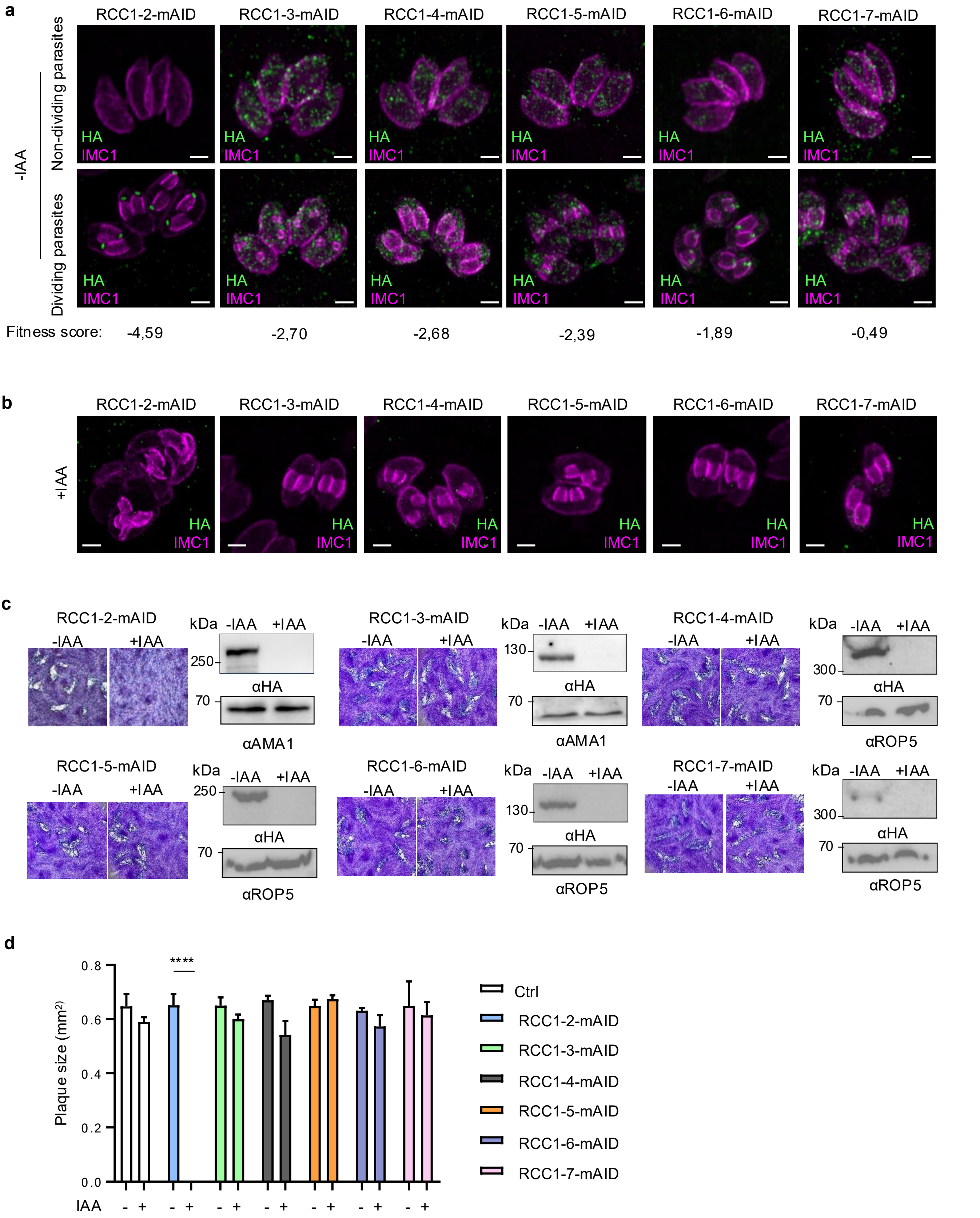
a
RCC1-2-mAID
RCC1-3-mAID
RCC1-4-mAID
RCC1-5-mAID
RCC1-6-mAID
RCC1-7-mAID
-IAA
Non-dividing parasites
Dividing parasites
HA
IMC1
Fitness score:
-4,59
-2,70
-2,68
-2,39
-1,89
-0,49
b
+IAA
c
kDa
-IAA
+IAA
250
130
300
70
αHA
αAMA1
αROP5
d
Plaque size (mm²)
0.8
0.6
0.4
0.2
0.0
****
IAA
Ctrl
RCC1-2-mAID
RCC1-3-mAID
RCC1-4-mAID
RCC1-5-mAID
RCC1-6-mAID
RCC1-7-mAID

bioRxiv preprint doi: https://doi.org/10.64898/2026.02.13.705759; this version posted February 14, 2026. The copyright holder for this preprint (which was not certified by peer review) is the author/funder, who has granted bioRxiv a license to display the preprint in perpetuity. It is made available under aCC-BY 4.0 International license.

Supplementary Figure 2. Conditional depletion of RCC1-repeats candidates. a, Confocal immunofluorescence images of the six RCC1-repeats containing proteins fused with a mAID-HA cassette (-mAID for short) in non-dividing (upper panel) and dividing parasites (lower panel). Parasites were stained with anti-HA (green) and anti-IMC1 (magenta) antibodies to visualize HA-tagged proteins and the IMC, respectively. Scale bars are 2μM. **b,** Same as **a**, in dividing parasites upon IAA depletion. Scales bars are 2μM. **c-d,** Assessment of fitness and protein depletion of the six RCC1-repeats containing proteins. **c,** Representative images of plaque assays for mAID-HA_3 tagged lines in the absence or presence of IAA and their corresponding immunoblot analysis, using an anti-HA antibody on lysates from intracellular parasites (mAID-HA tagged lines) that were either untreated or treated with IAA for 24h. TgAMA1 or TgROP5 served as a loading control. Protein molecular weight (in kDa) are shown on the left of each panel. **D,** Quantification of plaques areas for control (Tir1) and mAID-HA_3 tagged lines in the absence or presence of IAA. Values are reported as mean ± SD (n=3 biological replicates, each with three technical replicates). Statistical significance was determined by unpaired two-tailed Student's t-test. RCC1-repeats containing proteins were rapidly degraded upon addition of IAA and depletion resulted in no visible plaque formation after 7 days only for RCC1-2.

a

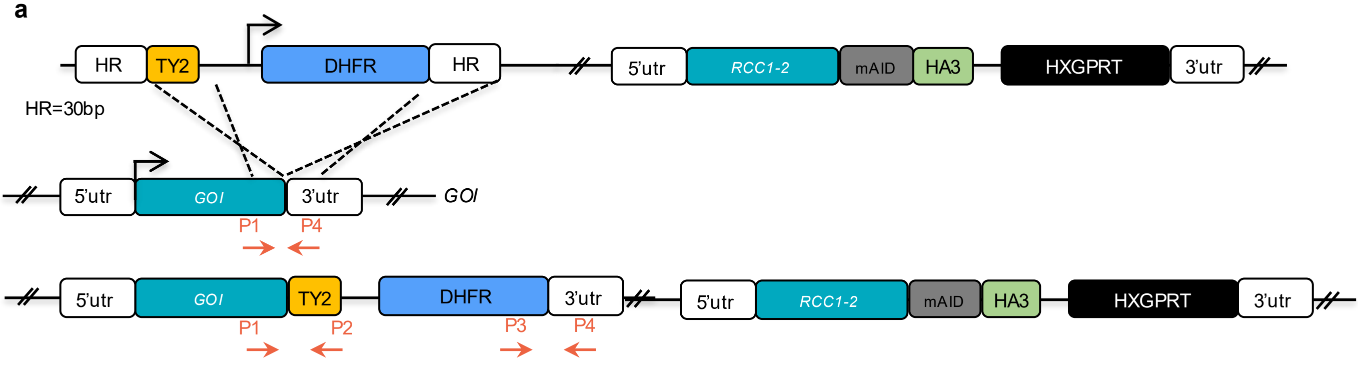

b

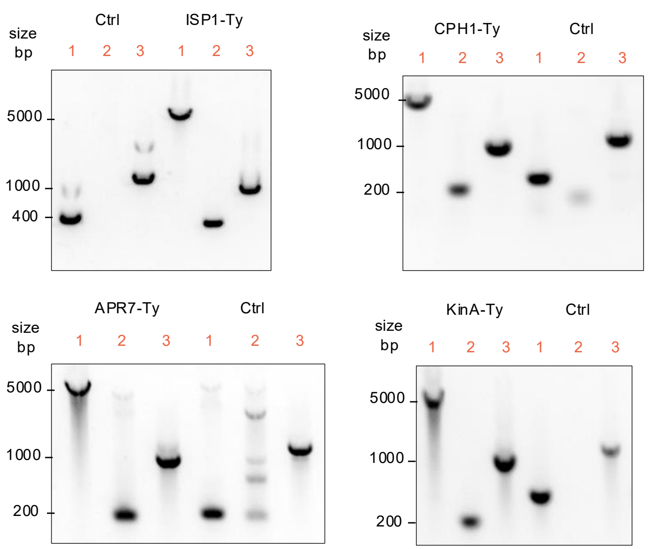

	ISP1	CPH1	APR7	KinA
1: P1+P4	4914bp	4745bp	4724bp	4758bp
2 : P1+P2	408bp	189bp	226bp	191bp
3 : P3+P4	936bp	985bp	927bp	996bp
Ctrl P1+P4	501bp	336bp	244bp	438bp

c

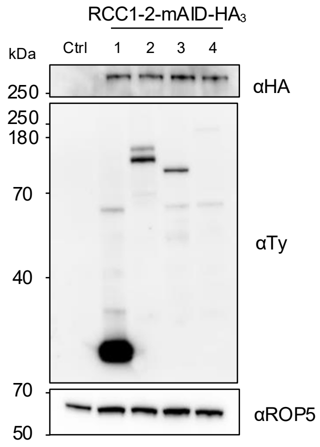

Lanes:
1: RCC1-2-mAID-HA$_3$ + ISP1-Ty2
2: RCC1-2-mAID-HA$_3$ + CPH1-Ty2
3: RCC1-2-mAID-HA$_3$ + APR7-Ty2
4: RCC1-2-mAID-HA$_3$ + KinA-Ty2

d

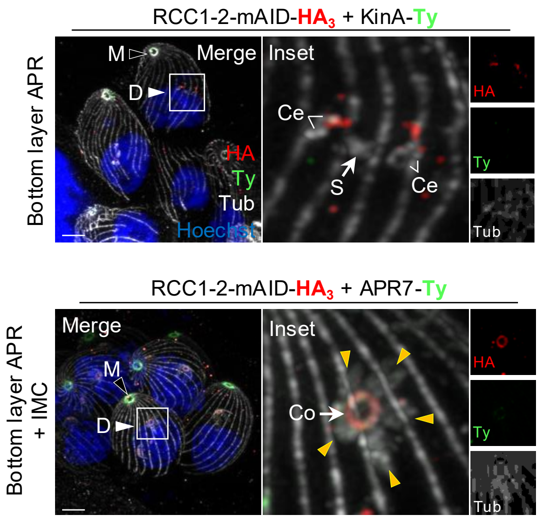

bioRxiv preprint doi: https://doi.org/10.64898/2026.02.13.705759; this version posted February 14, 2026. The copyright holder for this preprint (which was not certified by peer review) is the author/funder, who has granted bioRxiv a license to display the preprint in perpetuity. It is made available under aCC-BY 4.0 International license.

Supplementary Figure 3. Generation of double-tagged strains in RCC1-mAID-HA$_3$ strain. a, Illustration of the cloning strategy employed to obtain the double epitope-tagged RCC1-2-mAID-HA$_3$ strain using CRISPR/Cas9. Orange arrows depicted the designed primers used to validate the successful integration at the endogenous locus. **b,** Validation of successful integration of the TY2-DHFR cassette into *TgISP1*, *TgCPH1*, *TgAPR7* and *TgKinA* locus by PCR within the RCC1-2-mAID-HA$_3$ background. The parental strain Tir1 served as a negative control (Ctrl). Table lists expected PCR product sizes for each primer pair. **c,** Validation of successful expression of ISP1-Ty2, CPH1-Ty2, APR7-Ty2 and KinA-Ty2 in RCC1-2-mAID-HA$_3$ background. Immunoblot analysis using an anti-HA and anti-Ty antibodies was performed on lysates from intracellular parasites. TgROP5 was used as loading control. Protein molecular weights (in kDa) are indicated on the left of each panel. **d,** U-ExM images showing RCC1-2-mAID-HA$_3$ in combination with either KinA-Ty2 or APR7-Ty2 at early stage of endodyogeny. Intracellular tachyzoites were stained with anti-HA (red), anti-Ty (green) and anti-α/β tubulin (white) antibodies to label RCC1-2, apical markers and microtubules, respectively. Nuclear DNA was labeled with Hoechst (blue). Images represent maximum-intensity projections of z-stack confocal sections. Insets highlight developing daughter cells. White-contoured arrowheads indicate mother apical complex (M: mother). White arrowheads point developing daughter cells structures (D: daughter**)** (Ce: centrioles; S: spindle; Co: conoid). Yellow arrowheads mark the five rafts of nascents SPMTs. Scale bars are 5μM.

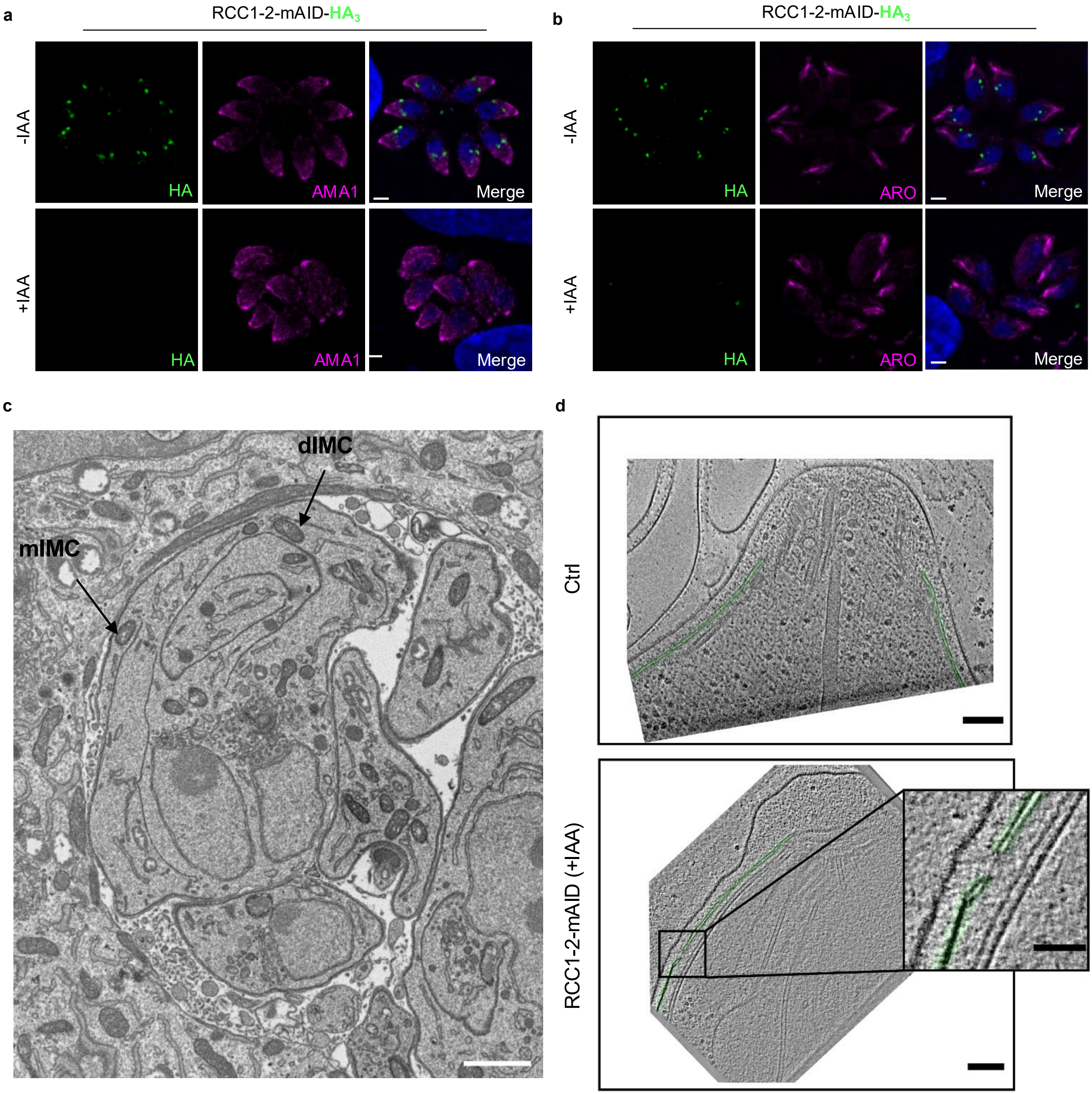
a
RCC1-2-mAID-HA3
-IAA
+IAA
HA
AMA1
Merge
b
RCC1-2-mAID-HA3
-IAA
+IAA
HA
ARO
Merge
c
dIMC
mIMC
d
Ctrl
RCC1-2-mAID (+IAA)

bioRxiv preprint doi: https://doi.org/10.64898/2026.02.13.705759; this version posted February 14, 2026. The copyright holder for this preprint (which was not certified by peer review) is the author/funder, who has granted bioRxiv a license to display the preprint in perpetuity. It is made available under aCC-BY 4.0 International license.

Supplementary Figure 4. RCC1-2 is essential for the ultrastructure organization and not for apical secretory organelles positionning. a-b, Confocal immunofluorescence images of RCC1-2-mAID-HA_3 parasites co-labeled for apical secretory organelles. Parasites were labeled with anti-HA (red) to label RCC1-2 and with either anti-AMA1 for micronemes **(a)** or anti-ARO for rhoptries **(b)** (green). Nuclear DNA was labeled with Hoechst (blue). Upper and lower panels show untreated and IAA-treated parasites for 24 hours, respectively, presented as maximum-intensity projections of z-stack confocal sections. Scale bars are 2µM. **c,** Representative electron micrographs of dividing RCC1-2-depleted tachyzoites. Black arrows highlight interrupted mother IMC (mIMC). Scale bars are 1µm. **d,** IMC Continuity**.** Tomogram slice demonstrates the preservation of the IMC (green) in WT parasites with intact plasma membrane **(**left panel). In contrast, RCC1-2 iKD (right panel**)** results in focal breakage and loss of structural continuity along the IMC (green) in parasites with intact PM, showing disintegration from the double membrane structure into a highly dense single membrane structure (inset). Scale bars: 100 nm.

Supplementary Fig. 5

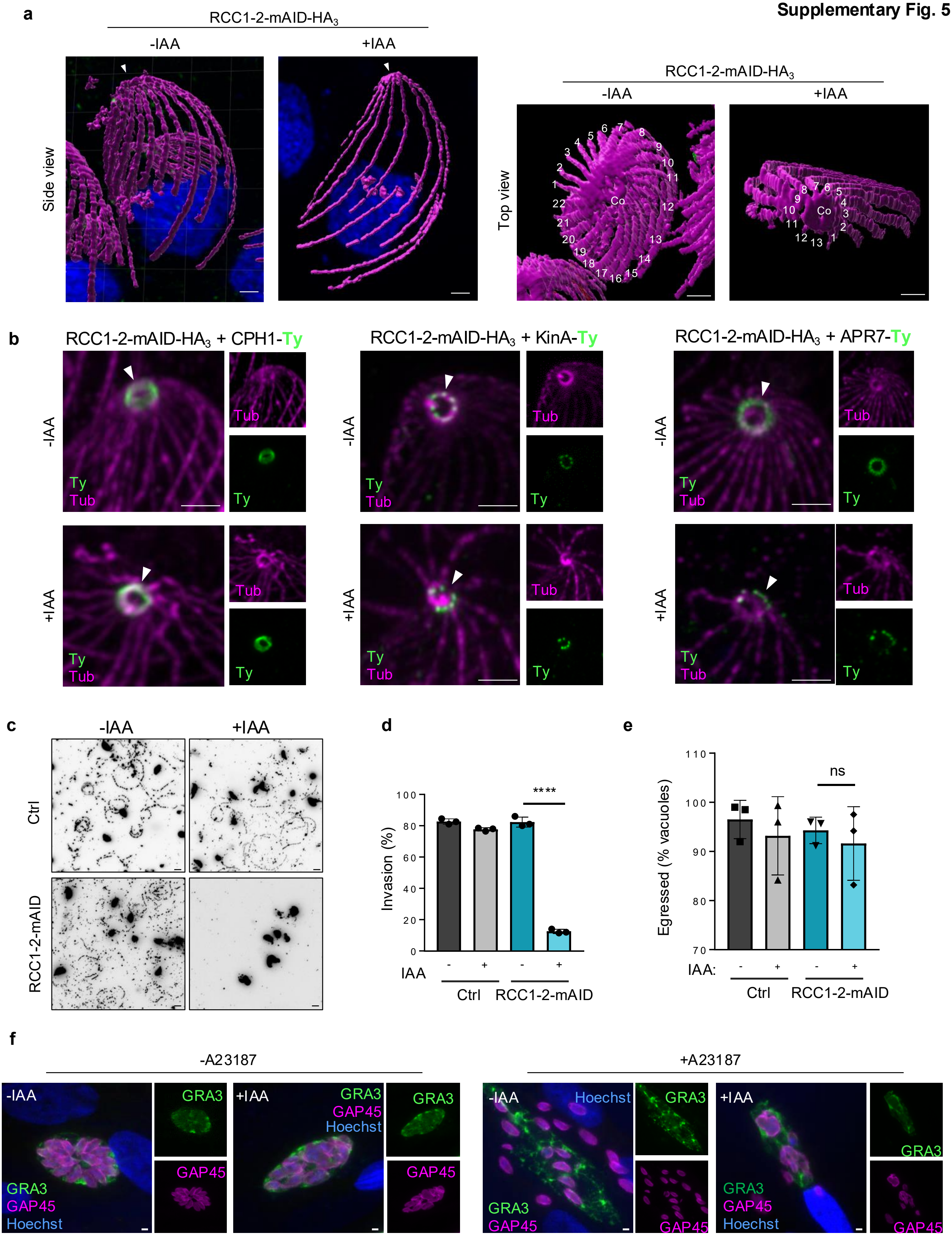

bioRxiv preprint doi: https://doi.org/10.64898/2026.02.13.705759; this version posted February 14, 2026. The copyright holder for this preprint (which was not certified by peer review) is the author/funder, who has granted bioRxiv a license to display the preprint in perpetuity. It is made available under aCC-BY 4.0 International license.

Supplementary Figure 5. Ultrastructural defects of RCC1-2 depleted parasites impede parasite motility and invasion. a, Imaris 3D reconstruction of U-ExM images from syringed-lysed tachyzoites, either left untreated or IAA-treated for 24h. Tachyzoites were stained with anti-α/β tubulin (magenta) antibody and Hoechst (blue) to label microtubules and nuclear DNA, respectively. Images represent maximum-intensity projections of z-stack confocal sections with both side and top views (left and right panels, respectively). White arrows indicate mother apical complex. SPMT numbers annotated on the top-view tachyzoite images. Scale bars are 1µM. **b,** Representative images of apical markers in mother cells by U-ExM. U-ExM images of RCC1-2-mAID-HA_3 + CPH1-Ty2 **(f)**, + KinA-Ty2 **(g)** and + APR7-Ty2 **(h)** parasites, either left untreated or treated with IAA for 24h. Intracellular tachyzoites were stained with anti-Ty (green) and anti-α/β tubulin (magenta) antibodies to label Ty-tagged proteins and microtubules, respectively. Images represent maximum-intensity projections of z-stack confocal sections. White arrows indicate mother apical complex. Scale bars are 2µM. RCC1-2 depletion leads to loss of APR7 integrity. **c,** Gliding motility is impaired upon RCC1-2 depletion: Parasites were treated with A23187 to stimulate gliding motility visualized by gliding trails labeled with an SAG1 antibody. Scale bars are 5µM. **d,** RCC1-2 depletion is responsible of an invasion defect: Invasion was monitored for control (Tir1) and RCC1-2-depleted tachyzoites upon 24 h treatment with IAA. Values are reported as mean ± SD ($n = 3$ biological replicates, each with three technical replicates). Statistical significance was determined by unpaired two-tailed Student's t-test. **e,** Stimulated parasite egress is not affected in the absence of RCC1-2: Infected cells were treated with A23187 to stimulate parasite egress, measured as a number of egressed parasitophorous vacuoles over the total number. Egress was tested for control (Tir1) and RCC1-2-mAID-HA_3 cell lines in the absence of IAA, and upon 24h of IAA treatment. Values are reported as mean ± SD ($n = 3$ biological replicates, each with three technical replicates). Statistical significance was determined by unpaired two-tailed Student's t-test. At least 100

bioRxiv preprint doi: https://doi.org/10.64898/2026.02.13.705759; this version posted February 14, 2026. The copyright holder for this preprint (which was not certified by peer review) is the author/funder, who has granted bioRxiv a license to display the preprint in perpetuity. It is made available under aCC-BY 4.0 International license.

PVs were quantified in each replicate. P-values are non-significant for all datasets (two-tailed t-test). **f,** Representative images of egress assay treated as in **e**. Parasites were stained with anti-GAP45 (red) and anti-GRA3 (green) antibodies to label parasites plasma membrane and parasitophorous vacuoles, respectively. DNA is labeled with Hoechst (blue). Scale bars are 2μM.

Supplementary Fig. 6

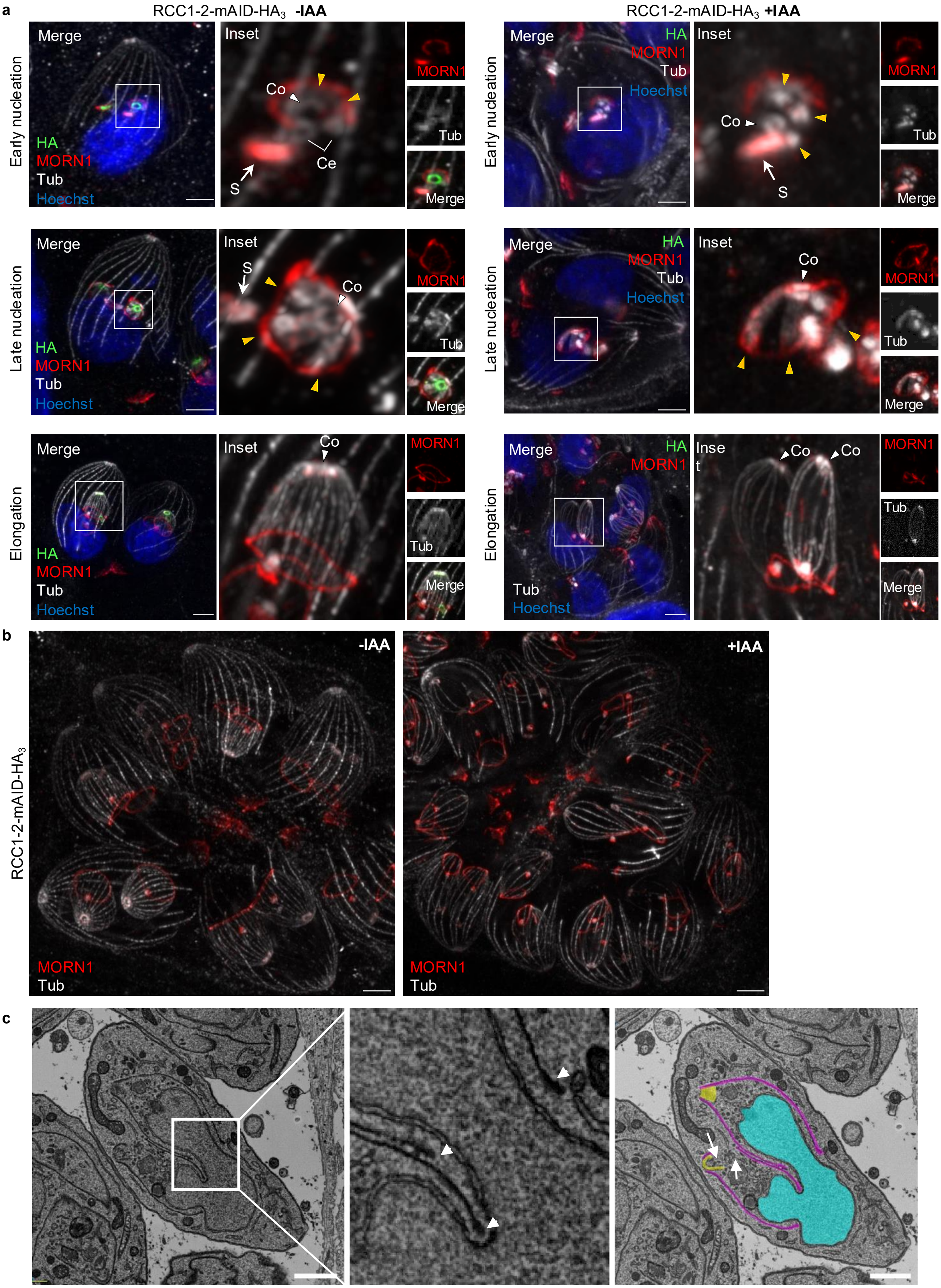

bioRxiv preprint doi: https://doi.org/10.64898/2026.02.13.705759; this version posted February 14, 2026. The copyright holder for this preprint (which was not certified by peer review) is the author/funder, who has granted bioRxiv a license to display the preprint in perpetuity. It is made available under aCC-BY 4.0 International license.

Supplementary Figure 6. RCC1-2 depletion leads to misshapen basal complex. a, Representative U-ExM images of RCC1-2-mAID-HA_3 upon later endodyogeny, left untreated or IAA-treated for 24h. Intracellular tachyzoites were labeled with anti-HA (green), anti-MORN1 (HA) and anti-α/β tubulin (white) antibodies to label RCC1-2, the basal complex and microtubules, respectively. Nuclear DNA was labeled with Hoechst (blue). Insets highlight developing daughter cells inside mother cell. Images represent maximum-intensity projections of z-stack confocal sections. White arrowheads point developing daughter cells structures (D: daughter**)** (Ce: centrioles; S: spindle; Co: conoid). Yellow arrowheads mark the rafts of nascents SPMTs. Scale bars are 5µM. RCC1-2 depletion leads to misshapen basal complex. **b,** Same as **a,** but showing widefield views with basal complex (red) and microtubules (white) labelling. Scale bars are 5µM. **c,** Representative electron micrographs of dividing RCC1-2-depleted tachyzoites. Left panel, with an inset zoom on one basal end, highlights the basal complex of the daughter cells IMC (white arrowhead). Right panel: Image segmentation shows the IMC in pink, the nucleus in blue, and conoid in yellow. The white arrow indicates a daughter IMC break. Scale bars are 1µm.

a

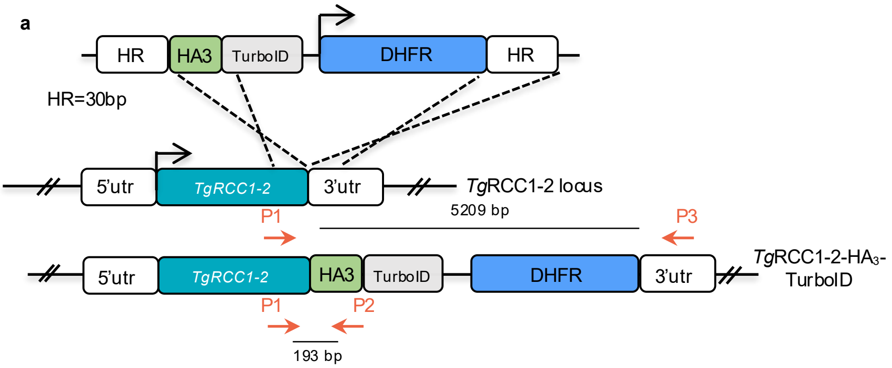

b

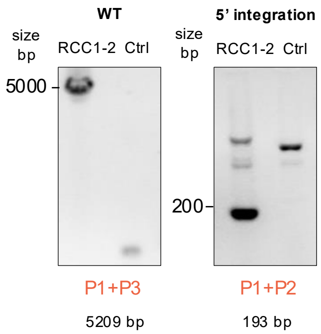

c

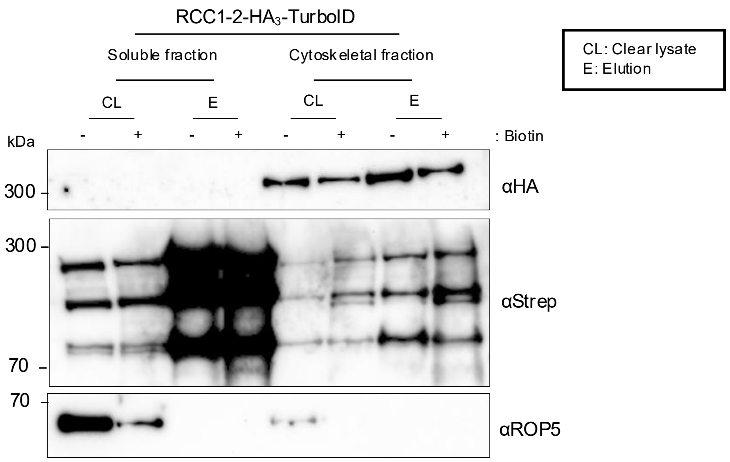

d

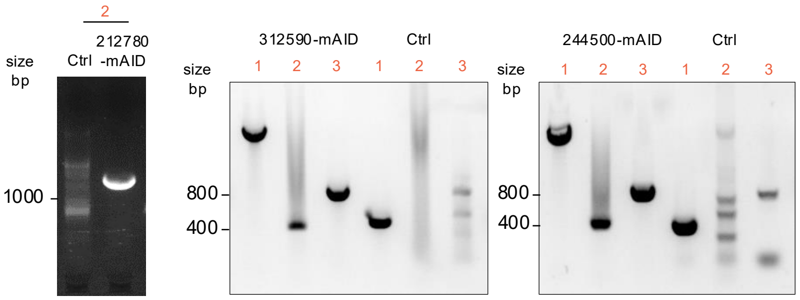

	212780	312590	244500
1: P1+P4	/	3035bp	3042bp
2 : P1+P2	1242bp	523bp	384bp
3 : P3+P4	/	758bp	815bp
Ctrl P1+P4	/	427bp	381bp

e

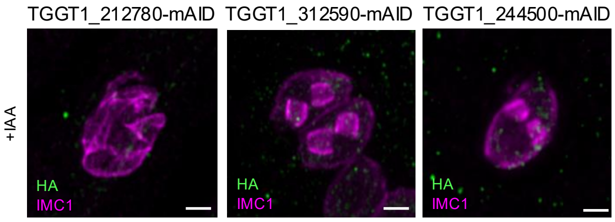

bioRxiv preprint doi: https://doi.org/10.64898/2026.02.13.705759; this version posted February 14, 2026. The copyright holder for this preprint (which was not certified by peer review) is the author/funder, who has granted bioRxiv a license to display the preprint in perpetuity. It is made available under aCC-BY 4.0 International license.

Supplementary Figure 7. Identification of new daughter-specific proteins by RCC1-2 proximity labelling. a, Illustration of the strategy employed to obtain the RCC1-2-HA_3-TurboID strain using CRISPR/Cas9. Orange arrows depicted the designed primers used to validate the successful integration at the endogenous loci with the expected PCR product sizes. **b,** Validation of successful integration of the HA_3-TurboID cassette into *TgRCC1-2* (*TGGT1_280770)* locus by PCR. The parental strain TaTi served as a negative control (Ctrl). **c,** Western blot analysis of clear lysates (CL) and eluted proteins (E) from RCC1-2-HA_3-TurboID parasites untreated or treated with biotin for 2 hours. Biotinylated proteins from soluble and cytoskeletal fractions were isolated with anti-streptavidin beads. Samples were immunoblotted with anti-HA (upper panel), anti-streptavidin-HRP (middle panel). TgROP5 was used as loading control (lower panel). RCC1-2 was detected only in the cytoskeletal fraction. **d,** Validation of successful integration of the mAID-HA_3 cassette (-mAID for short) into *TGGT1_212780, TGGT1_312590, TGGT1_244500* locus by PCR. The parental strain Tir1 served as a negative control (Ctrl). Table lists expected PCR product sizes for each primer pair. **e,** Confocal immunofluorescence images of the three RCC1-2 proximity candidates fused with a mAID-HA cassette (-mAID for short) in IAA-treated parasites for 24 hours. Parasites were stained with anti-HA (green) and anti-IMC1 (magenta) antibodies to visualize HA-tagged proteins and the IMC, respectively. Scale bars are 2μM.

Supplementary Fig. 8

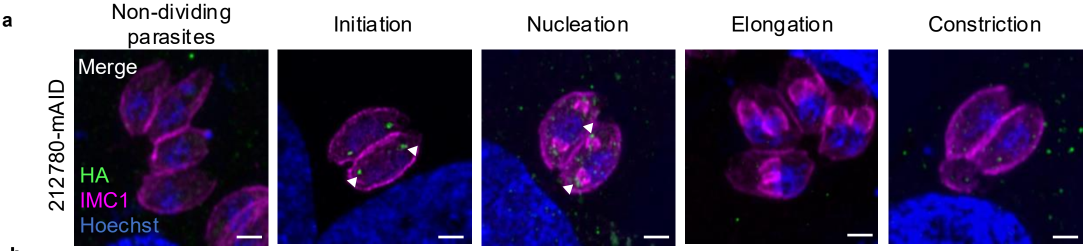

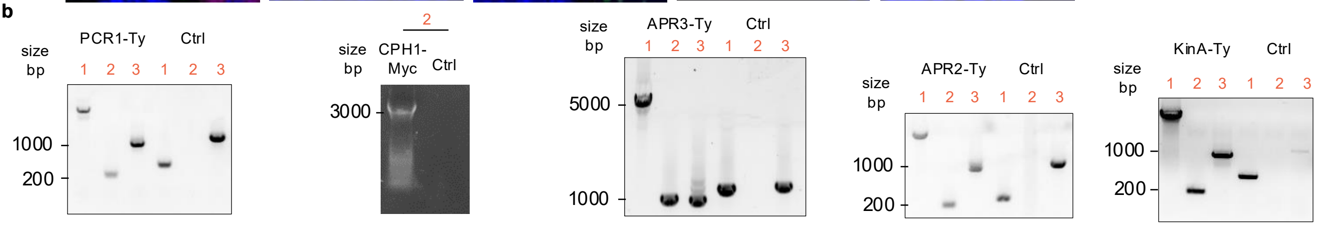

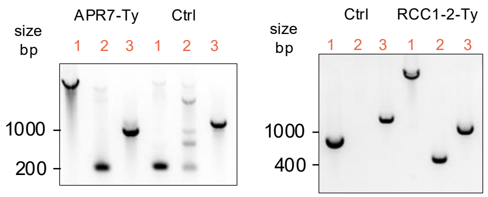

	PCR1	CPH1	APR3	APR2	KinA	APR7	RCC1-2
1: P1+P4	4774bp	/	5573bp	4788bp	4758bp	4724bp	5026bp
2 : P1+P2	239bp	3452bp	1015bp	231bp	191bp	226bp	466bp
3 : P3+P4	964bp	/	987bp	986bp	996bp	927bp	989bp
Ctrl P1+P4	400bp	/	1153bp	307bp	438bp	244bp	663bp

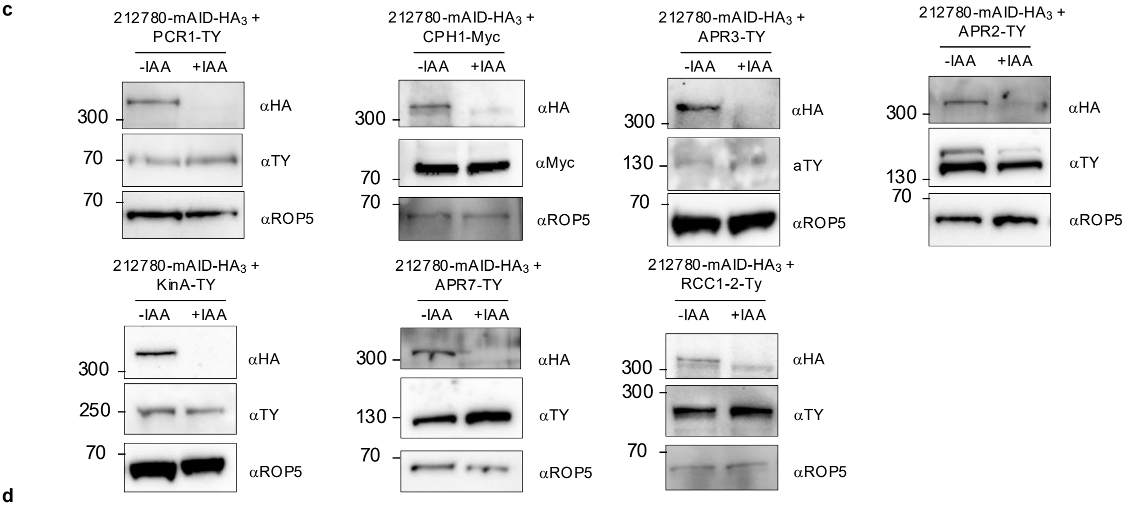

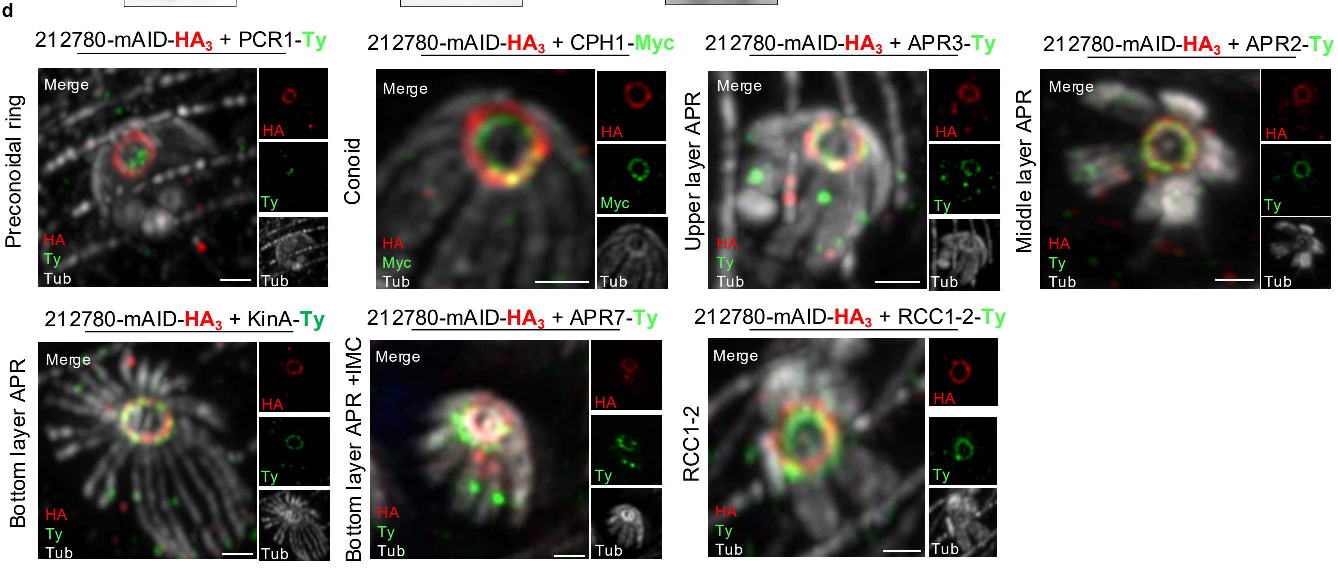

e

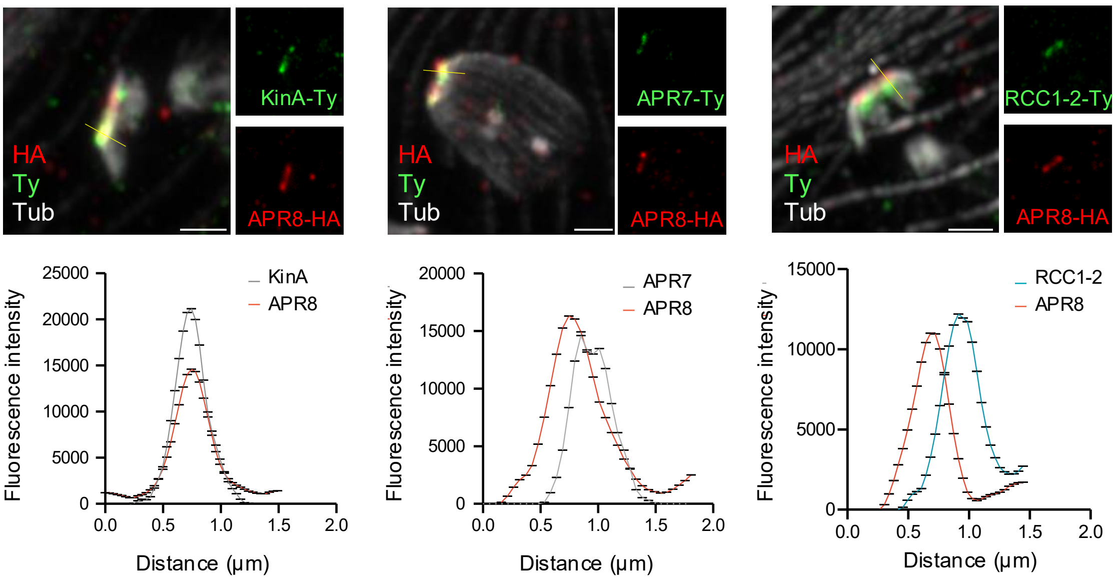

f

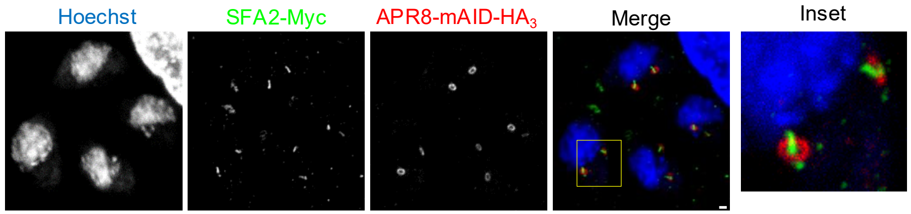

g

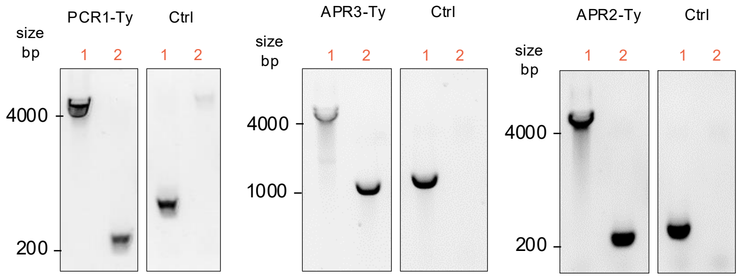

APR8-mAID-HA3 + CPH1-Myc

	PCR1	APR3	APR2
1: P1+P4	4774bp	5573bp	4788bp
2 : P1+P2	239bp	1015bp	231bp
Ctrl P1+P4	400bp	1153bp	307bp

h

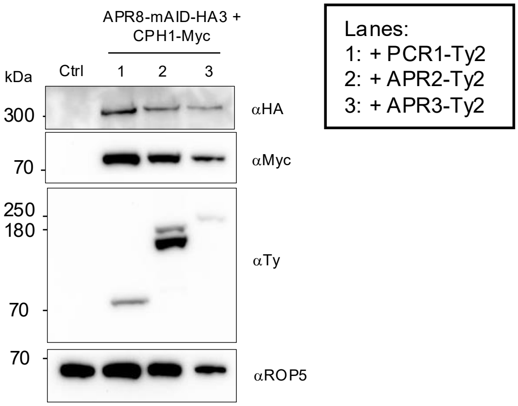

bioRxiv preprint doi: https://doi.org/10.64898/2026.02.13.705759; this version posted February 14, 2026. The copyright holder for this preprint (which was not certified by peer review) is the author/funder, who has granted bioRxiv a license to display the preprint in perpetuity. It is made available under aCC-BY 4.0 International license.

Supplementary Figure 8. Generation of double and triple-tagged strains in TGGT1_212780-mAID-HA$_3$ strain. a, Confocal immunofluorescence images of TGGT1_212780-mAID-HA$_3$ localization. Parasites were labeled with anti-HA (green) and anti-IMC1 (magenta) antibodies to visualize TGGT1_212780 and the IMC, respectively. DNA is labeled with Hoechst. White arrowheads point at TGGT1_212780 signal. Shown are maximum-intensity projections of z-stack confocal images. Scale bars are 2μM. **b,** Validation of successful integration of the MYC2-DHFR cassette into *TgCPH1* locus and TY2-DHFR cassette into *TgPCR1*, *TgAPR3*, *TgAPR2*, *TgKinA*, *TgAPR7* and *TgRCC1-2* locus by PCR within the TGGT1_212780-mAID-HA$_3$ background. The parental strain Tir1 served as a negative control (Ctrl). Table lists expected PCR product sizes for each primer pair. **c,** Assessment of protein expression and depletion of the TGGT1_212780-mAID-HA$_3$ co-expressing either Ty-tagged or Myc-tagged apical complex proteins. Immunoblot analysis using an anti-HA, anti-Ty or anti-Myc antibodies was performed on lysates from intracellular parasites that were either left untreated or treated with IAA for 6h. TgROP5 was used as loading control. Successful insertional tagging of CPH1-Myc2, PCR1-Ty2, APR3-Ty2, APR2-Ty2, KinA-Ty2, APR7-Ty2 and RCC1-2-Ty2 was verified and their expression levels remained unchanged upon RCC1-2 depletion. Protein molecular weights (in kDa) are indicated on the left of each panel. **d,** U-ExM images of TGGT1_212780-mAID-HA$_3$ in combination with multiple Ty/Myc-tagged apical complex proteins, shown as top view of a single daughter cell. Intracellular tachyzoites were stained with anti-HA (red), anti-Ty or anti-Myc (green) and anti-α/β tubulin (white) antibodies to label TGGT1_212780, apical markers and microtubules, respectively. Images represent maximum-intensity projections of z-stack confocal sections. Scale bars are 2μM. **e,** Colocalization of APR8-mAID-HA3 with Ty-tagged proteins KinA, APR7 and RCC1-2 by U-ExM. Intracellular tachyzoites were stained with anti-HA (red), anti-Ty (green) and anti-α/β tubulin (white) antibodies to label APR8, Ty-tagged proteins and

bioRxiv preprint doi: https://doi.org/10.64898/2026.02.13.705759; this version posted February 14, 2026. The copyright holder for this preprint (which was not certified by peer review) is the author/funder, who has granted bioRxiv a license to display the preprint in perpetuity. It is made available under aCC-BY 4.0 International license.

microtubules, respectively. The direction used to measure the fluorescence intensity in daughter cells, which generates the colocalization curve, is indicated by the yellow bar. Scale bars are 1µM. **f,** U-ExM images of APR8-mAID-HA_3 in combination with the Myc-tagged protein SFA2. Intracellular tachyzoites were stained with anti-HA (red), anti-Myc (green) antibodies to label APR8 and the striated fiber component SFA2, respectively. Nuclear DNA was labeled with Hoechst (blue). Images represent maximum-intensity projections of z-stack confocal sections. Scale bars are 1µM. **g,** Validation of successful integration of the TY2-DHFR cassette into *TgPCR1, TgAPR3* and *TgAPR2* locus by PCR into the APR8-mAID-HA_3 + CPH1-My2 background. The parental strain Tir1 served as a negative control (Ctrl). Table lists expected PCR product sizes for each primer pair. **h,** Validation of successful expression of PCR1-Ty2, APR3-Ty2 and APR2-Ty2 in APR8-mAID-HA_3 + CPH1-Myc2 background. Immunoblot analysis using an anti-HA, anti-Ty and anti-Myc antibodies was performed on lysates from intracellular parasites. TgROP5 was used as loading control. Protein molecular weights (in kDa) are indicated on the left of each panel.

Supplementary Fig. 9

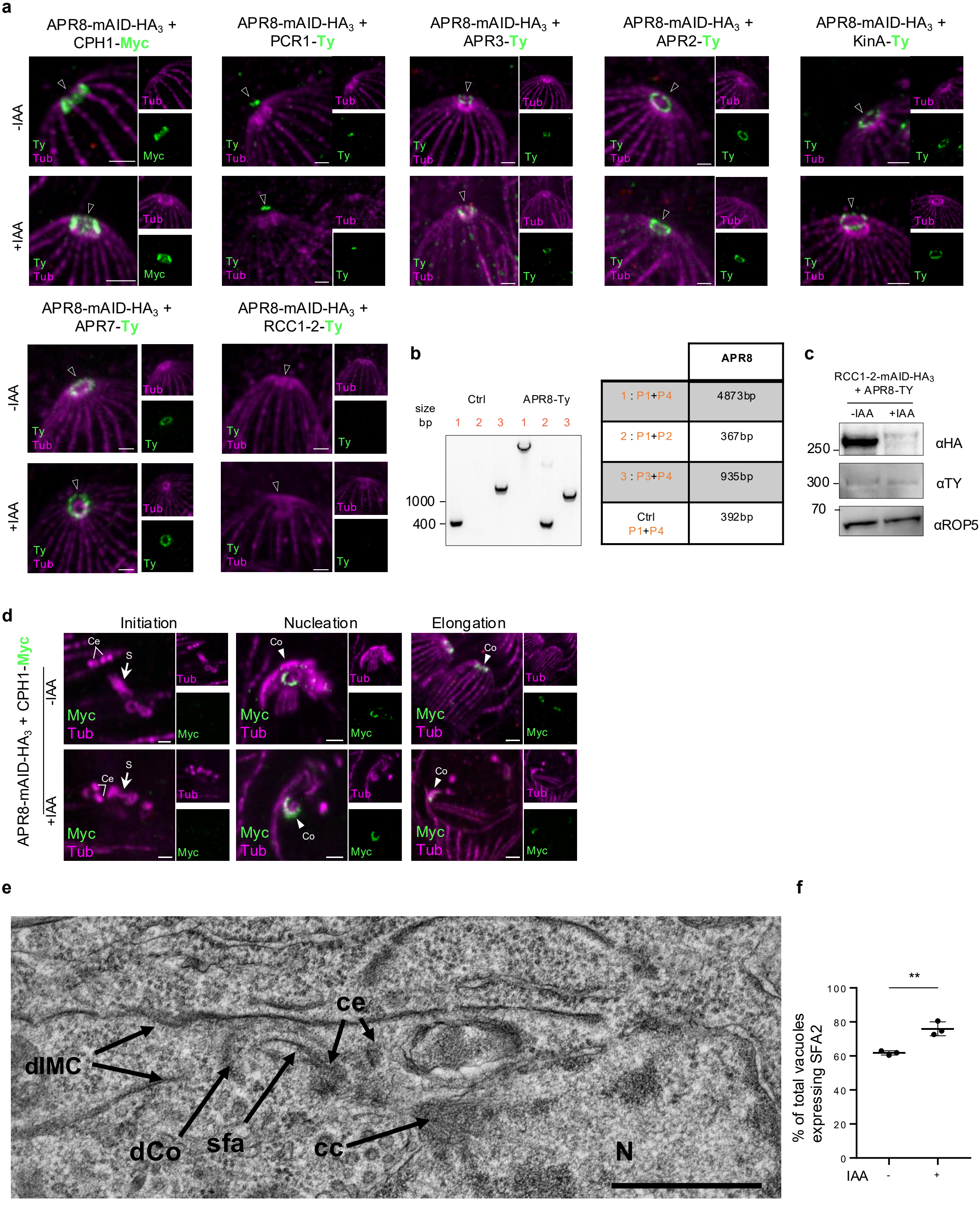

bioRxiv preprint doi: https://doi.org/10.64898/2026.02.13.705759; this version posted February 14, 2026. The copyright holder for this preprint (which was not certified by peer review) is the author/funder, who has granted bioRxiv a license to display the preprint in perpetuity. It is made available under aCC-BY 4.0 International license.

Supplementary Figure 9. APR8 depletion impairs APR organization. a, Representative U-ExM images of mother apical complex in APR8-mAID-HA_3 strain with Ty-/Myc-tagged apical markers, either left untreated or IAA-treated for 6h. Intracellular tachyzoites were stained with anti-Ty or anti-Myc (green) and anti-α/β tubulin (magenta) antibodies to label apical markers and microtubules, respectively. Images represent maximum-intensity projections of z-stack confocal sections, with inset zoom highlighting a single mother apical complex. White arrowheads point mother conoid. Scale bars are 1μM. **b,** Validation of successful integration of the TY2-DHFR cassette into *TgAPR8* locus by PCR into the RCC1-2-mAID-HA_3 background. The parental strain Tir1 served as a negative control (Ctrl). Table lists expected PCR product sizes for each primer pair. **c,** Assessment of protein expression and depletion of the RCC1-2-mAID-HA_3 co-expressing Ty-tagged APR8. Immunoblot analysis using an anti-HA and anti-Ty antibodies was performed on lysates from intracellular parasites that were either left untreated or treated with IAA for 24h. Validation of successful expression of APR8-Ty2 in RCC1-2-mAID-HA_3 background. Immunoblot analysis using an anti-HA and anti-Ty antibodies was performed on lysates from intracellular parasites. TgROP5 was used as loading control. Protein molecular weights (in kDa) are indicated on the left of each panel. **d,** U-ExM images of APR8-mAID-HA_3 + CPH1-Myc2 from initiation to elongation stage of endodyogeny. Intracellular parasites were either left untreated or IAA-treated for 4h. Parasites were stained with anti-Myc (green) and anti-α/β tubulin (magenta) antibodies to label the conoid protein CPH1 and microtubules, respectively. Images represent maximum-intensity projections of z-stack confocal sections. Insets highlight developing daughter cells. White arrowheads point developing daughter cells structures (Ce: centrioles; S: spindle; Co: conoid). Scale bars are 1μM. **e,** Representative transmission electron microscopy (TEM) images of APR8-depleted tachyzoites. Black arrows highlight nascent daughter conoid (dCo) is still connected to centrioles (ce) via the striated fiber (sfa). (dIMC: daughter IMC; cc: centrocone;

bioRxiv preprint doi: https://doi.org/10.64898/2026.02.13.705759; this version posted February 14, 2026. The copyright holder for this preprint (which was not certified by peer review) is the author/funder, who has granted bioRxiv a license to display the preprint in perpetuity. It is made available under aCC-BY 4.0 International license.

daughter IMC; cc: centrocone; N: nucleus). Scale bars are 0,5 µm. **f,** Quantification of SFA labeling by indirect immunofluorescence analysis in APR8-mAID-HA3 parasites, left untreated or IAA-treated for 6 hours. Vacuoles were quantified from approximately 100 vacuoles and are presented as mean ± SD (n = 3 biological replicates). Statistical significance was determined by unpaired two-tailed Student's t-test.

bioRxiv preprint doi: https://doi.org/10.64898/2026.02.13.705759; this version posted February 14, 2026. The copyright holder for this preprint (which was not certified by peer review) is the author/funder, who has granted bioRxiv a license to display the preprint in perpetuity. It is made available under aCC-BY 4.0 International license.

Supplementary movie 1: Imaris 3D reconstruction of U-ExM images from intracellular RCC1-2-mAID-HA3 parasites. Parasites were stained with anti-HA (green) and anti-α/β tubulin (magenta) antibodies to label RCC1-2 and microtubules, respectively. Nuclear DNA is labelled with Hoechst. RCC1-2 and microtubules were segmented using the Surface module. Scale bars are 10μM. Synchronous division occurs in rosette-patterned parasitophorous vacuoles and RCC1-2 localized at the apical end of developing daughter cells.

Supplementary movie 2: Imaris 3D reconstruction of U-ExM images from intracellular RCC1-2-mAID-HA3 parasites upon IAA depletion. Parasites were stained with anti-HA (green) and anti-α/β tubulin (magenta) antibodies to label RCC1-2 and microtubules, respectively. Nuclear DNA is labelled with Hoechst. Microtubules were segmented using the Surface module. Scale bars are 10μM. Tachyzoites undergo asynchronous division and presents SPMTs defects.

Supplementary movie 3: Imaris 3D reconstruction of U-ExM images from extracellular RCC1-2-mAID-HA3 parasites. Parasites were stained anti-α/β tubulin (magenta) antibodies and Hoechst to label microtubules and nuclear DNA, respectively. Microtubules were segmented using the Surface module. Scale bars are 5μM. Tachyzoites appears as elongated crescent shape with 22 SPMTs anchored at the APR.

Supplementary movie 4: Imaris 3D reconstruction of U-ExM images from extracellular RCC1-2-mAID-HA3 parasites upon IAA depletion. Parasites were stained anti-α/β tubulin (magenta) antibodies and Hoechst to label microtubules and nuclear DNA, respectively. Microtubules were segmented using the Surface module. Scale bars are 5μM. The symmetry

bioRxiv preprint doi: https://doi.org/10.64898/2026.02.13.705759; this version posted February 14, 2026. The copyright holder for this preprint (which was not certified by peer review) is the author/funder, who has granted bioRxiv a license to display the preprint in perpetuity. It is made available under aCC-BY 4.0 International license.

and organization of SPMTs in tachyzoites is disrupted in absence of RCC1-2. Parasites lacked the full complement of 22 SPMTs and loss their elongated crescent shape.

Supplementary movie 5: Imaris 3D reconstruction of U-ExM images from intracellular RCC1-2-mAID-HA_3 parasites with basal complex labelling. Parasites were stained with anti-HA (green), anti MORN1 (red) and anti-α/β tubulin (white) antibodies to label RCC1-2, the basal complex and microtubules, respectively. Nuclear DNA is labelled with Hoechst. RCC1-2, basal complex and microtubules were segmented using the Surface module. Only one daughter cell was 3D-reconstructed to facilitate visualization of the connection between the basal complex and the SPMTs. Scale bars are 5µM. RCC1-2 localized to the apical end of daughter cell. MORN1 localized to the basal end of daughter cells as a ring structure connected to daughter SPMTs. Daughter cells form elongated domes within the mother cell

Supplementary movie 6: Imaris 3D reconstruction of U-ExM images from intracellular RCC1-2-mAID-HA3 parasites with basal complex labelling upon IAA depletion. Parasites were stained with anti-HA (green), anti MORN1 (red) and anti-α/β tubulin (white) antibodies to label RCC1-2, the basal complex and microtubules, respectively. Nuclear DNA is labelled with Hoechst. RCC1-2, basal complex and microtubules were segmented using the Surface module. Only one replicating mother cell was fully 3D-reconstructed to facilitate visualization of the SPMTs defects. Scale bars are 5µM. Daughter cells show flattened morphology upon RCC1-2 depletion, concomitant with SPMT shape defects. The corresponding ring structure observed for the basal complex is often irregular or appeared as discontinuous segments.
